# New Genome-Wide Algorithm Identifies Novel *In-Vivo* Expressed *Mycobacterium Tuberculosis* Antigens Inducing Human T-Cell Responses with Classical and Unconventional Cytokine Profiles

**DOI:** 10.1038/srep37793

**Published:** 2016-11-28

**Authors:** Mariateresa Coppola, Krista E. van Meijgaarden, Kees L. M. C. Franken, Susanna Commandeur, Gregory Dolganov, Igor Kramnik, Gary K. Schoolnik, Inaki Comas, Ole Lund, Corine Prins, Susan J. F. van den Eeden, Gro E. Korsvold, Fredrik Oftung, Annemieke Geluk, Tom H. M. Ottenhoff

**Affiliations:** 1 Department of Infectious Diseases, Leiden University Medical Center, Leiden, The Netherlands; 2 Department Microbiology Immunology, Stanford Univ. School of Medicine, Stanford, USA; 3 Department Immunology Infectious Diseases, Harvard School of Public Health, Boston, USA; 4 Institute of Biomedicine of Valencia (IBV-CSIC), Valencia, Spain; 5 CIBER in Epidemiology and Public Health, Madrid, Spain; 6 Dept. Systems Biology, Technical Univ., Denmark; 7 Department of Infectious Disease Immunology, Domain for Infection Control and Environmental Health, Norwegian Institute of Public Health, Oslo, Norway

## Abstract

New strategies are needed to develop better tools to control TB, including identification of novel antigens for vaccination. Such *Mtb* antigens must be expressed during *Mtb* infection in the major target organ, the lung, and must be capable of eliciting human immune responses. Using genome-wide transcriptomics of *Mtb* infected lungs we developed data sets and methods to identify IVE-TB (*in-vivo* expressed *Mtb*) antigens expressed in the lung. Quantitative expression analysis of 2,068 *Mtb* genes from the predicted first operons identified the most upregulated IVE-TB genes during *in-vivo* pulmonary infection. By further analysing high-level conservation among whole-genome sequenced *Mtb*-complex strains (*n* = 219) and algorithms predicting HLA-class-Ia and II presented epitopes, we selected the most promising IVE-TB candidate antigens. Several of these were recognized by T-cells from *in-vitro Mtb-*PPD and ESAT6/CFP10-positive donors by proliferation and multi-cytokine production. This was validated in an independent cohort of latently *Mtb*-infected individuals. Significant T-cell responses were observed in the absence of IFN-γ-production. Collectively, the results underscore the power of our novel antigen discovery approach in identifying *Mtb* antigens, including those that induce unconventional T-cell responses, which may provide important novel tools for TB vaccination and biomarker profiling. Our generic approach is applicable to other infectious diseases.

Since WHO in 1993 declared tuberculosis (TB) a global public health emergency, 22 million lives have been saved and 56 million patients have been adequately treated^1^. Despite these encouraging achievements, it will not be possible to eradicate TB solely by conventional strategies. Recent modelling studies^2^ show that the current decline in TB incidence needs to be accelerated significantly, which cannot be achieved without developing and implementing novel tools. The increase in multidrug-resistance incidence^3^, the association with HIV-infection and non-communicable diseases^4^, the lack of an efficient vaccine and the difficulty in timely diagnosing TB^5^ represent just a few of the challenges that need to be addressed. Improved diagnostics^6^, safer and more accessible treatments for active and latent *Mycobacterium tuberculosis (Mtb*) infection^7^ and new effective pre- and post-exposure vaccines must all be developed before we can reach TB elimination^8^,^9^.

Sixteen TB vaccine candidates are currently in various phases of clinical testing^10^. The majority of these candidates are subunit vaccines which mainly contain combinations of a limited number of antigens identified mostly under *in vitro* culture conditions, thereby representing less than one percent of the targetable ~4000 proteins encoded by *Mtb* genome^11^. So far, in humans, none of these vaccines has shown to confer protection superior to Bacillus Calmette-Guérin (BCG), the only licensed TB vaccine available. Intradermal BCG administration by itself, however, does not induce protection against TB consistently^12^,^13^.

Recently, MVA85A, the first single antigen-based TB vaccine candidate tested in an efficacy phase IIb trial, failed to boost protection against TB in BCG-vaccinated infants^14^. Following this disappointing outcome, the field has been reconsidering the design of future TB vaccines^15^, including new efforts towards better rationalized and comprehensive antigen selection approaches, both in pre-clinical^16^ and clinical studies^17^,^18^,^19^,^20^.

To identify new *Mtb* antigens, we have hypothesized it is important to consider host-pathogen interactions in antigen discovery, rather than focussing on the pathogen alone. We have proposed to define the *Mtb* antigen repertoire expressed *in vivo* during pulmonary infection. The lung is the primary organ in which *Mtb* resides and interacts with the human immune system^9^,^11^,^21^,^22^, and where it initiates the process of infection and pathogenesis, setting the stage for future transmission.

In our previous preliminary study^23^, we have explored gene expression profiles during pulmonary infection in mice with varying susceptibility to TB. In combination with the *in vitro* immune characterization of human cells from *Mtb* exposed individuals, this work allowed us to identify the first *in vivo* expressed TB candidate antigens (IVE-TB). This included Rv2034, which induced poly-functional T cell responses with *Mtb-*inhibitory activity at the clonal level^24^ and induced encouraging protection against TB challenge in HLA-DR transgenic mice and in guinea pigs^25^. Several other strategies have been followed to explore the *Mtb* anti-genome^11^, including predictive MHC-peptide binding bioinformatics tools combined with human or mouse based T-cell screening approaches to discover novel HLA-class Ia^11^,^26^, Ib^27^ and II^11^,^19^,^28^,^29^ restricted *Mtb* antigens.

In this study, we have used genome-wide transcriptomics of *Mtb* from the lungs of infected mice, and developed extensive data sets and methods to identify promising pulmonary IVE-TB antigens. We generated a novel *Mtb* gene expression database from isolated *Mtb* RNA from the lungs of large numbers of susceptible (C3HeB/FeJ) and resistant (C57BL/6J) mice after aerosol *Mtb* (Erdman) infection, and analysed the expression of 2,068 *Mtb* genes to allow the selection of the most upregulated IVE-TB genes during early and late phase infection. By combining these data with extended analysis of high-level genetic conservation in a set of 219 whole-genome sequenced *Mtb* complex strains^30^, and with the presence of HLA class Ia and II peptide binding motifs as well as homology with other pathogenic mycobacteria, new IVE-TB candidate antigens could be selected. Several of these antigens were recognized by T-cells from *in vitro Mtb* responsive donors and latently *Mtb-*infected donors as measured by proliferation and multi-cytokine production, including antigens that induced responses other than IFN-γ. Collectively, these results underscore the power of our novel antigen discovery approach in identifying new *Mtb* antigens which may provide important tools for TB vaccination and biomarker profiling studies.

## Results

### Identification of novel *in vivo* expressed *Mtb* (IVE-TB) genes upregulated during pulmonary *Mtb* infection

**Figure 1:**
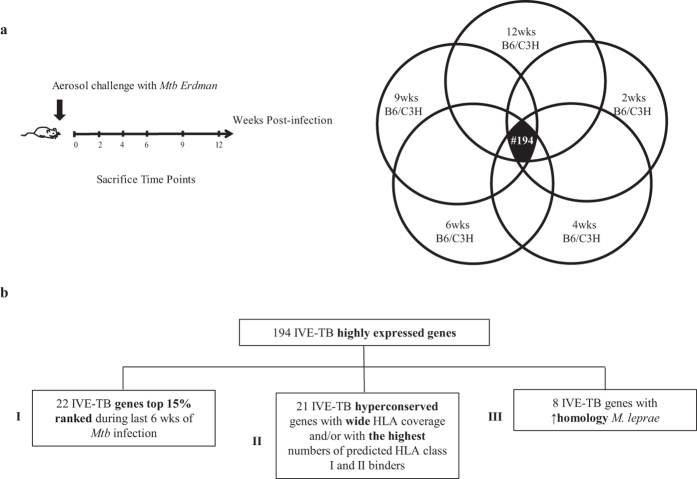
Gene expression analysis of IVE-TB genes during pulmonary infection and *In vivo* expressed *Mtb* (IVE-TB) gene selection algorithm. (**a**) The time line represents the timescale used to challenge and sacrifice at 2, 4, 6, 9 and 12 weeks (wks) TB resistant (Bl6) and susceptible (C3H) mice (*n* = 4 per group). For each time point*, Mtb* RNA was isolated from the lung of *Mtb*-infected mice and the expression of 2068 *Mtb* RGCNs was analysed. The most consistently upregulated IVE-TB genes (*n* = 194) during early and late phase murine pulmonary *Mtb* infection were selected independently from the host background. Each circle corresponds to the top 15% highest expressed *Mtb* genes per mouse strain and time point of *Mtb* infection. The overlapping area in black represents the 194 overlapping IVE-TB genes common to all conditions. (**b**) The flow chart shows the analyses performed to determine the most promising *in vivo* expressed (IVE-TB) *Mtb* antigens candidates from the 194 IVE-TB genes selected according to their high expression during murine pulmonary *Mtb* infection. Four subgroups were selected combining different parameters: I/gene expression during last 6 weeks (wks) of *Mtb* infection; II/conservation, wide HLA class Ia and II alleles coverage and/or the highest number of HLA-peptide binding motifs for HLA class I and II III/high homology with *M. leprae*. Genes that could not be expressed as recombinant proteins were excluded.

To discover antigens able to elicit cellular immune responses against *Mtb* proteins highly expressed in the lung, we investigated the *in vivo* expression profile of 2,068 *Mtb* genes (IVE-TB) based on *Mtb* RNA isolated from murine infected pulmonary tissue. IVE-TB qRT-PCR-based gene expression data sets were obtained from groups of four mice per strain, characterized by high vs. low susceptibility to tuberculosis (C3HeB/FeJ vs. C57BL/6J), at five time points (Fig. 1a) after aerosol *Mtb* Erdman infection. Data were normalized to standard reference gene copy numbers (RGCNs). IVE-TB genes with the highest and most persistent RGCNs were selected (top 15% expressed genes in all 10 data sets), independently of host background or infection phase, resulting in 194 candidates (Fig. 1a; Table S1). The proportional distribution of the functional categories among these 194 IVE-TB genes was compared to that of the whole *Mtb* genome. The categories *intermediary metabolism and respiration, regulatory protein, cell wall and cell processes, information pathways, lipid metabolism,* were over-represented among the IVE-TB genes; while the categories *virulence, detoxification, adaptation, PE/PPE, conserved hypotheticals* and *insertion seqs and phages* were under-represented (Fig. S1).

### Conservation and *in silico* predicted antigenicity of the top up-regulated IVE-TB genes

To upselect IVE-TB candidates for immunogenicity testing in humans, we characterised the most up-regulated 194 IVE-TB genes for: their level of conservation among 219 *Mtb* whole-genome sequences; for their homology with BCG and other pathogenic mycobacteria; and for their *in silico* predicted antigenicity.

First, all 194 selected IVE-TB genes were screened for their amino acid (aa) sequence homology with 219 whole-genome sequenced *Mtb* complex strains isolated from clinical specimens as described^30^. Considering the number of non-synonymous aa changes for each protein weighted for the number of aa, 154 genes were found to be highly conserved (aa conservation ≥98%) among all seven *Mtb* lineages (Table S2). Further BLASTp analysis showed that most of the IVE-TB protein sequences were highly homologous to proteins present in BCG and other pathogenic (*M. leprae, M. ulcerans*) or atypical mycobacteria (*M. kansasii*) (NTM) (Table S3). This observation suggests that the majority of IVE-TB genes potentially could boost specific immune responses induced by previous BCG vaccination or by NTM exposure.

Secondly, since classical HLA-restricted T-cell responses are required for protection against TB, two *in-silico* tools, NetMHCcons 1.0^31^ and NetMHCIIpan 3.0^32^, were used to predict HLA class Ia and class II binding epitopes, respectively, in the 194 IVE-TB gene-encoded proteins. This query provided both quantitative and qualitative data, i.e. the absolute number of predicted peptide binding motifs for any HLA class Ia or class II molecule as well as the HLA class Ia and class II alleles predicted to bind epitopes from the proteins, respectively (Table S2). The HLA alleles included in this analysis should provide a large global coverage of the human HLA class Ia and II polymorphisms [virtually 98% for HLA class Ia^33^ and approximately 50–75% for HLA class II]^34^.

### Upselection of candidate IVE-TB genes for immunogenicity testing on human cells

We next combined the *in vivo* expression data with the *in-silico* analysis to identify the most promising IVE-TB candidate antigens among the 194 upregulated IVE-TB genes (Fig. 1b, Table S4).

The first selected genes (*n* = 22) (subgroup I) were those that ranked as top 15% IVE-TB genes during the later time points of *Mtb* infection (6 wks), since we hypothesised this to be an important parameter in upselecting antigens for driving T-cell responses against *Mtb* in the infected lung.

The second selection (*n* = 21) (subgroup II) included hyperconserved IVE-TB genes with wide HLA class Ia and class II allele coverage and/or with the highest number of peptide epitopes predicted to bind to HLA class Ia and HLA class II. The last group (*n* = 8) (subgroup III) of IVE-TB genes consisted of *Mtb* genes with high homology to *M. leprae* (genes taxid: 1769) (Rv0468, Rv0470c, Rv0501, Rv0640, Rv1390, Rv1846, Rv1872 and Rv2215). The rationale behind the last selection was to include candidates with potential cross-protection against *M. leprae* infection^35^,^36^. In addition, since these genes are conserved in *M. leprae*, which has strongly downsized its genome size compared to other pathogenic mycobacteria, these *M. leprae* genes could play important roles in intracellular mycobacterial survival^37^. One gene (Rv0287) overlapped between two subgroups (I and II).

In view of high-throughput recombinant protein production, some genes, although matching the criteria of inclusion, had to be excluded based on aa length and/or predicted transmembrane regions and hydrophobicity, leading to expression and solubility issues. Two additional genes (Rv1197 and Rv1805) (from subgroups I and II) were deselected due to the low yields obtained during recombinant protein production (Table S4). Combining all three subgroups, a total of 50 genes was upselected for further evaluation.

The most represented functions of the 50 IVE-TB genes were in line with the categories over-represented in the 194 IVE-TB genes compared to the whole *Mtb* genome. Some of the 50 IVE-TB genes selected here have already been described as T cell antigens and have been formulated as TB vaccines currently in clinical trials^20^,^29^,^38^,^39^,^40^ or pre-clinical studies^16^,^23^,^26^,^28^,^41^,^42^ (Table S4). Those consistent findings strongly validate our antigen discovery algorithm. For comparative purposes, the known antigens matching our selection procedure were included in the immunogenicity study below.

ESAT6 was part of the 50 IVE-TB genes fulfilling the inclusion criteria and was produced as fusion protein dimerized to CFP10 (E/C), since this is known to enhance its discriminatory TB diagnostic value^6^,^43^. Another five esx genes qualifying as IVE-TB were also formulated as heterodimers with their co-expressed partner proteins. The two IVE-TB gene pairs Rv0287/Rv0288 and Rv2346/Rv2347 were produced as fusion proteins (Rv0287/88 and Rv2346/47). Rv3615 was produced and tested as single protein as well as heterodimer with Rv3614 (although Rv3614 was not in the IVE-TB gene selection).

Taken together, 49 recombinant proteins was produced for antigenicity testing. Since E/C was used as a diagnostic to distinguish previous exposure to *Mtb* we analysed this fusion protein separately, such that we refer in the below to a total of 48 IVE-TB proteins for further analyses.

### Recognition of the novel IVE-TB antigens by human T cell proliferation

As a first step in evaluating the newly selected IVE-TB proteins (*n* = 48), we tested their immunogenicity using PBMCs from individuals with or without pre-existing immune response to mycobacteria (*n* = 19). Based on their *in vitro* proliferative responses to *Mtb* ESAT6/CFP10 (E/C) and *Mtb* purified protein derivative (PPD), donors were divided into three subgroups: double responders (E/C^+^and PPD^+^), single responders (E/C^+^or PPD^+^) and non-responders (E/C^−^ and PPD^−^) to *Mtb*.

**Figure 2:**
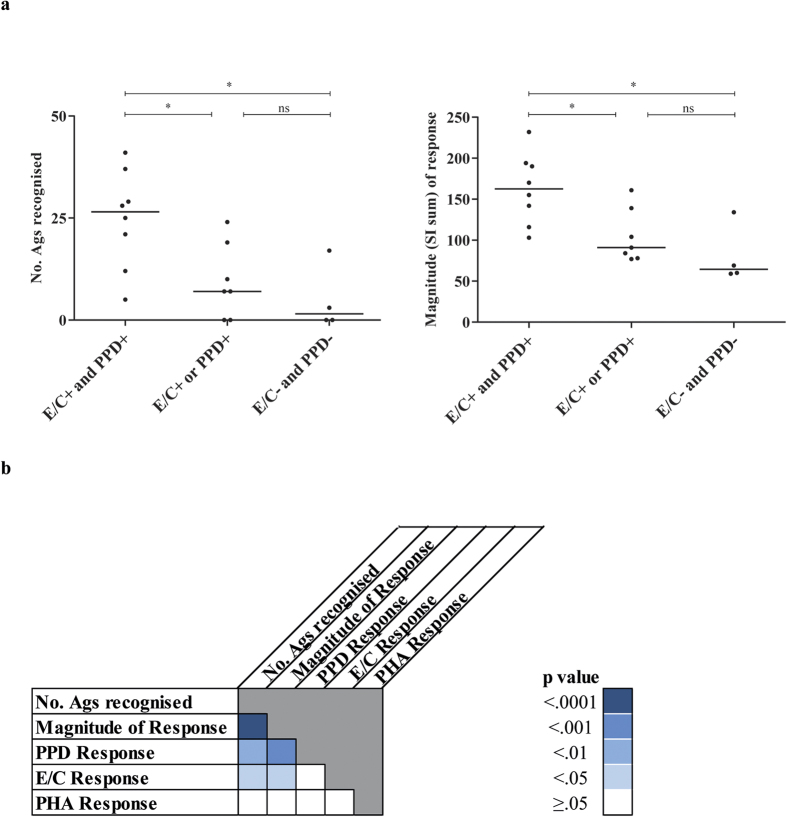
Lymphocyte proliferative responses to IVE-TB proteins in individuals with different exposures to *Mtb*. (**a**) Donors (*n* = 19) were divided into three groups based on their lymphocyte proliferative response to *Mtb* ESAT6/CFP10 (E/C) or PPD. The total number of antigens recognised (left) and the overall magnitude of responses generated (right) after stimulation are indicated per individual as a closed circle (•). (**b**) Donors were ranked according to the lymphocyte proliferation responses to PHA, PPD, E/C, the total proliferation, and the number of antigens recognised. The correlation among these variables was measured by Spearman r, and the statistical significance was expressed by two-tailed p-value.

The double responders recognised many antigens (range = 5–41 out of the 48 selected antigens) in contrast to single responders (range = 0–24 antigens) or non-responders groups (range = 0–17 antigens) (p = 0.02). A similar trend was observed for the magnitude of the proliferative response to the antigens: when expressed as stimulation index (SI), values ranged from 103 to 232 in the double responders, from 77 to 161 in the single responders and from 59 to 134 in the non-responders (Fig. 2a) (p = 0.02). Interestingly, the number of antigens recognised and the magnitude of response produced by each donor were strongly correlated (Spearman correlation: r = 0.96; p < 0.0001) (Fig. 2b) indicating both high immunogenicity and immunodominance. The significantly greater T-cell proliferation observed in the double responders compared to the single or non-responder groups correlated with the presence of *Mtb* orthologues in NTM or BCG. We have previously reported that more than 50% of healthy donors from our Northern EU, non-endemic area responded to PPD^23^,^41^. This is further supported here by the strong correlation observed between the magnitude of the response to known mycobacterial stimuli (PPD, E/C) and the magnitude of response to the IVE-TB antigens (PPD: Spearman correlation r = 0.72, p < 0.001; E/C: Spearman correlation r = 0.48, p < 0.05) as well as the number of IVE-TB antigens recognised in relation to the donors *Mtb* responder status (PPD: Spearman correlation r = 0.64, p < 0.01; E/C: Spearman correlation r = 0.53, p < 0.05) (Fig. 2b).

**Figure 3:**
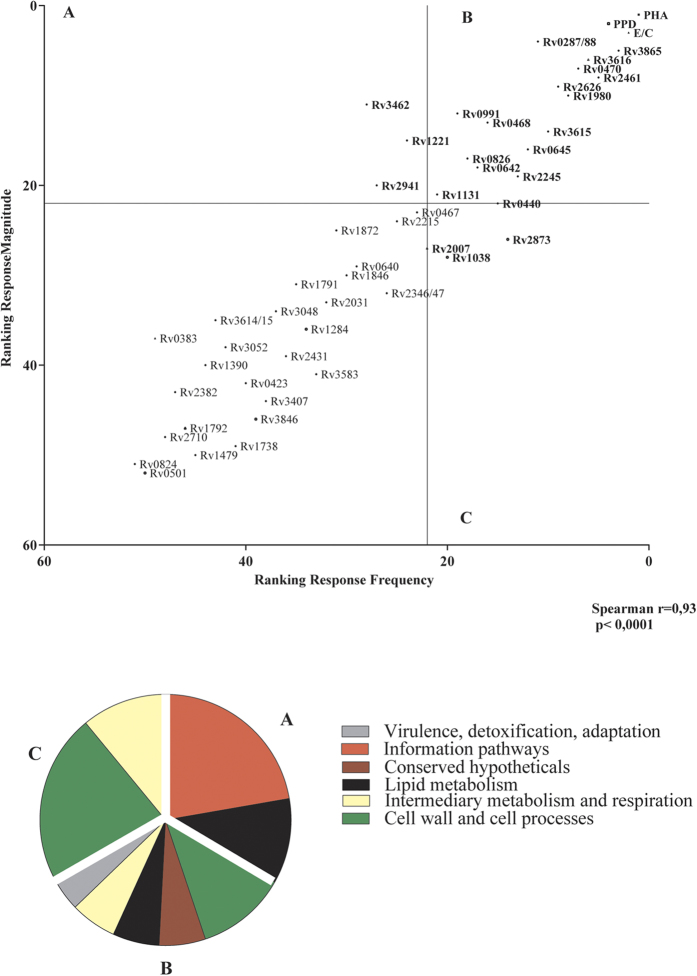
Immunodominance and immunogenicity of IVE-TB antigens based on lymphocyte proliferative responses. The immunodominance (x-axis) and immunogenicity (y-axis) of the new IVE-TB antigens (Ags), based on antigen-specific T cell proliferation measured in the single (E/C+ or PPD+) and double (E/C+ and PPD+) responders (n = 15), were compared. The correlation was determined by Spearman r, and the statistical significance was calculated as a two-tailed p-value. In quadrant A are the top immunogenic antigens, in quadrant C the top frequently recognized antigens, and in quadrant B the top antigens for both parameters. The pie chart shows the distribution of the functional categories for each quadrant of the graph.

Next, we compared the relative immunodominance and the immunogenicity of the 48 IVE-TB antigens and controls (E/C, PPD, and PHA) based on SI values measured in the single (E/C+ or PPD+) and double (E/C+ and PPD+) responders (n = 15). By ranking the antigens according to the number of positive responses (immuno-dominance) and the magnitude of proliferation elicited by each antigen for all donors (immunogenicity), we found that these parameters correlated well (Spearman correlation r = 0.93, p < 0.0001). Using this distribution it was possible to identify a group of 16 from the top 20 antigens significantly concordant for both variables. Their functions did not only fall into categories overrepresented among the 194 IVE-TB genes (*intermediary metabolism and respiration, cell wall and cell process, information pathways* and *lipid metabolism*), but also in one underrepresented category (*virulence, detoxification and adaptation*) (Fig. 3).

In summary, these results show that from the 48 tested IVE-TB proteins, 16 were frequently and highly recognised by the PBMCs from donors with recall responses to mycobacteria as indicated by *Mtb* specific T cell proliferation.

### Cytokine responses to the new IVE-TB antigens induced from PBMC of *Mtb*-ESAT6/CFP10 and/or PPD *in vitro* responders

Next, to investigate the immunogenicity in more depth and characterize the type of T cell responses involved, we evaluated the induction of cytokines triggered by the IVE-TB proteins (*n* = 48) in the same donors (*n* = 12) evaluated in the above proliferation assays. PBMCs were stimulated with each IVE-TB antigen, E/C, PPD or PHA for six days and culture supernatants were tested for the presence of IFN-γ, IP-10, TNF-α, IL-17, IL-13, IL-10 and GM-CSF using a seven-plex assay. IL-10 was excluded from further analysis due to the low concentrations measured in response to antigens and PHA.

**Figure 4:**
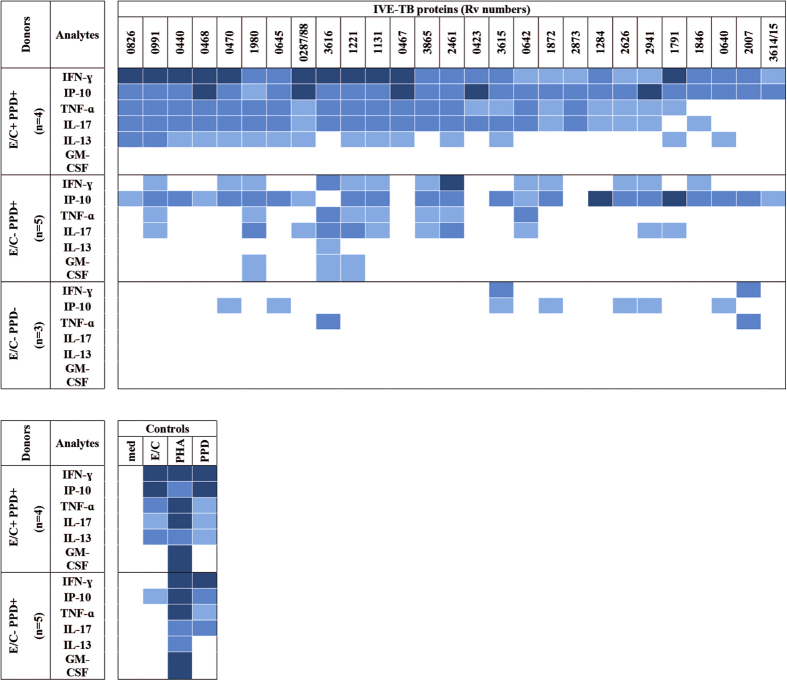
Cytokine production in response to IVE-TB antigens in PBMC of individuals differently exposed to mycobacteria. PBMCs of ESAT/CFP10 (E/C) and PPD *in vitro* positive (+) or negative (−) donors were stimulated with each IVE-TB antigen, E/C, PPD or PHA for six days. The culture supernatants were tested for the presence of IFN-γ, IP-10, TNF-α, IL-17, IL-13, IL-10 and GM-CSF using a seven-plex assay. Results are shown for those IVE-TB antigens which elicited at least two different cytokines in ≥50% of the donors. Percentage of responders: 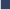 = 100%, 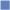 = 75–85% and 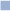 = 50–60%.

First, we analysed the number of individuals responding to the IVE-TB antigens based on the induced levels of the different cytokines. As in our previous report, we used IFN-γ concentrations ≥100 pg/ml in response to PPD and E/C, to discriminate individuals previously exposed to NTM or *Mtb* respectively; according to this criterion, donors were subsequently again divided into three groups: E/C^+^ PPD^+^, E/C^−^ PPD^+^, and E/C^−^ PPD^−^. As already described elsewhere^44^,^45^,^46^, IFN-γ secretion did not always correlate with proliferative activity, suggesting that alternative cytokine responses might be involved. Half of the E/C^+^ PPD^+^ donors produced at least two different cytokines in response to 27 out of 48 IVE-TB proteins. Thirteen out of these 27 IVE-TB antigens induced increased levels of not only IFN-γ and IP-10 but also of TNF-α, IL-17 and IL-13. In the E/C^−^ PPD^+^ group, higher levels of at least two different cytokines were induced by 15 IVE-TB antigens, which overlapped with those described for the E/C^+^ PPD^+^ group, although with a slightly different cytokine pattern than the double positive responders group. In fact, TNF-α, IL-17, and IL-13 were induced with lower frequencies in the E/C^−^ PPD^+^ group, while GM-CSF was induced with higher frequencies by three antigens (Rv1221, Rv1980 and Rv3616c). In the E/C^−^ PPD^−^ group, double cytokine induction was observed for only two out of the 27 antigens (Rv3615, and Rv2007) (Fig. 4).

**Figure 5:**
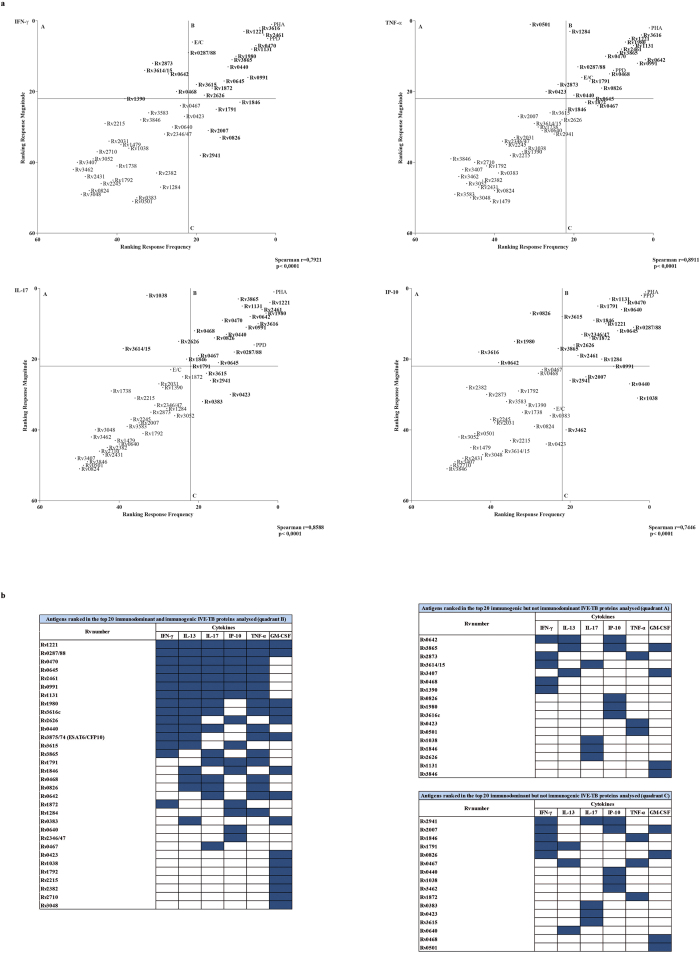
Immunodominance and immunogenicity of IVE-TB antigens based on cytokine production. PBMCs of *Mtb*-responders (*n* = 9) were stimulated with IVE-TB antigens and tested for the levels of multiple cytokines by multiplex assay. (**a**) The frequency (x-axis) and the magnitude (y-axis) of responses were ranked to compare the immunodominance and the immunogenicity of each antigen, respectively, per analyte as well as for the responses to the positive controls (PPD and PHA). In quadrant A are the top antigens for immunogenicity, in quadrant C the top antigens for immunodominance, and in quadrant B the top antigens for both parameters. Results were determined by Spearman’s rank correlation coefficient. Graphs are shown for IFN-γ, IL-17, TNF-α and IP-10 as examples. (**b**) Summary of the top immunogenic and/or immunodominant 20 IVE-TB antigens (present in quadrant A, B or C) for all the cytokines analysed.

Subsequently, for each analyte the frequency and the magnitude of IVE-TB antigen induced responses were ranked to establish relative immunodominance and immunogenicity based on cytokine profiles. A significant correlation between these parameters was found for all cytokines analysed (Spearman correlation with p < 0.0001for: r^IFN-γ^ = 0.80, r^IP-10^ = 0.74, r^TNF-α^ = 0.89, r^IL-17^ = 0.86, r^IL-13^ = 0.87, r^GM-CSF^ = 0.74) (Fig. 5a and Fig. S2). Of note, several IVE-TB antigens were found to be both immunogenic and immunodominant for most cytokines considered (Fig. 5b).

**Figure 6:**
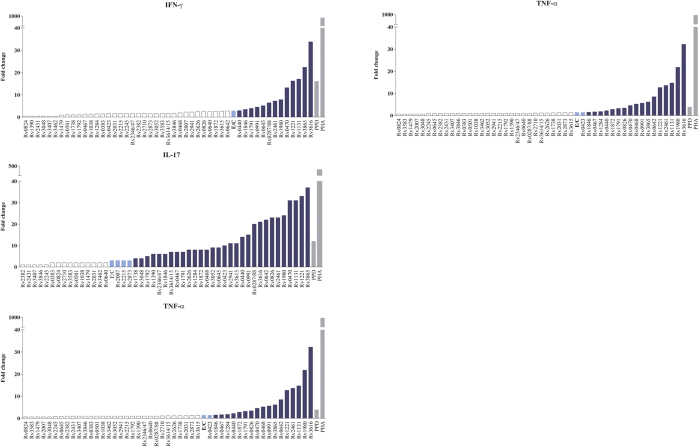
Comparison of cytokine production elicited by E/C with that induced by IVE-TB antigens. The fold change in the median cytokine levels of stimulated vs. unstimulated samples from the *Mtb*-exposed individuals (*n* = 12) was calculated for ESAT6/CFP10 (E/C), IVE-TB antigens (*n* = 50) and controls (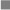). Results are shown only for IFN-γ, IL-17, TNF-α and IP-10. The cytokine fold changes induced by the IVE-TB antigens were either similar to (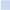), greater than (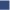), or less than (□) those induced by E/C.

To compare the level of cytokines induced by each IVE-TB antigen with that produced by E/C, the fold change in the median cytokine levels of stimulated vs. unstimulated samples from the *Mtb*-exposed individuals (double or single responders) was calculated. Notably, distinct cytokine profiles were observed for different antigens (Fig. 6; Table S5).

In conclusion, most of the IVE-TB antigens inducing cytokine responses (*n* = 27) were recognised by *Mtb*-exposed individuals by the induction of several cytokines, with responses highly correlated in terms of magnitude and frequency. For cytokines other than IFN-γ, the magnitude of the reactivity to the IVE-TB antigens differed from that to E/C. Importantly, many cytokines other than IFNγ were induced. Especially pro-inflammatory and Th1- related IP-10, TNF, IL-17 and also Th2 related IL-13 revealed a much wider response pattern than expected.

### Cytokine responses to novel IVE-TB antigens in an independent cohort of latently *Mtb-*infected individuals

**Figure 7:**
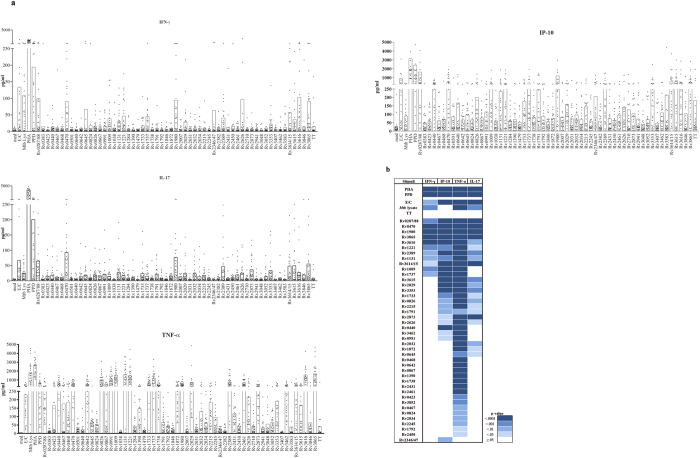
Cytokine response profile to IVE-TB antigens in LTBI. The cytokine levels were measured in diluted whole blood supernatants of an independent cohort of LTBI (*n* = 25) after 6 days stimulation with either IVE-TB antigens (10 μg/ml), ESAT6/CFP10 (E/C) (10 μg/ml), PPD (5 μg/ml), PHA (2 μg/ml) or tetanus toxoid (TT) (10 μg/ml). (**a**) Bars represent the mean of responses. Results are shown for IFN-γ, IL-17, TNF-α and IP-10 as illustrative examples. (**b**) Significance differences were evaluated between the cytokine level induced by IVE-TB antigens vs. unstimulated (Dunn’s multiple comparisons test).

To validate these results in an independent cohort, we evaluated the immunogenicity of the selected IVE-TB proteins in latently *Mtb-*infected (LTBI) donors (*n* = 25). For comparative purposes we also included ten additional *Mtb* antigens identified previously (Rv0867, Rv1009, Rv1733, Rv1737, Rv2029, Rv2032, Rv2034, Rv2389, Rv2450, and Rv3353)^23^,^24^,^41^,^47^,^48^,^49^,^50^,^51^,^52^,^53^,^54^. Diluted whole blood samples were stimulated for six days and the cytokine levels were assessed by a seven-plex assay (data shown only for IFN-γ, IL-17, TNF-α and IP-10 Fig. 7a). Significant differences (Dunn’s multiple comparisons test) were observed between the unstimulated and the stimulated samples in the levels of all analytes except for IL-13, IL-22, and IL-9 (Fig. 7b). Forty-two out of the 58 antigens significantly increased the production of at least one cytokine, particularly TNF-α. Remarkably, several antigens induced distinct cytokines simultaneously. In this regard, E/C, PHA, PPD and 11 antigens (Rv0287/88, Rv0470, Rv1009, Rv1737, Rv1980, Rv3865, Rv3616, Rv1221, Rv2389, Rv1131, and Rv3614/15) induced substantial and concomitant secretion of IFN-γ, IP-10 and TNF-α, sometimes in combination with IL-17. Notably, several antigens did not induce IFN-γ but nevertheless induced other cytokines. In fact, nine antigens (Rv3615, Rv2029, Rv3353, Rv1733, Rv0826, Rv2215, Rv1791, Rv2873, and Rv2626) simultaneously increased IP-10, TNF-α and IL-17 secretion in the absence of significant IFN-γ. Six antigens (Rv0440, Rv3462, Rv0991, Rv2031, Rv1872, and Rv0645) induced high production of TNF-α in combination with either IL-17 or IP-10 in the absence of significant IFN-γ. No specific association between known functions of the *Mtb* proteins and the pattern of cytokine expression could be distinguished (Fig. S3).

Collectively, the results demonstrate that this independent cohort of LTBI donors recognised most of the selected IVE-TB antigens as judged by the induction of multiple cytokines, confirming and extending the immunogenicity of the IVE-TB antigens uncovered in this study.

## Discussion

The urgent quest for *Mtb* antigens capable of inducing protective immunity against *Mtb*^16^ prompted us to develop a new algorithm to identify novel immunogenic *Mtb* proteins. Several studies have pursued strategies to discover new *Mtb* peptide epitopes and their related antigens using *in silico* predictive tools and functional immunomic approaches^26^,^28^,^55^. However, as far as we are aware, our approach focusing on *in vivo* expressed (IVE-TB) *Mtb* genes^23^ is unique, because it concentrates on real-time *in vivo Mtb* pulmonary infection based gene expression data. Our rationale is that effective *Mtb* antigens need to be expressed in the main target organ of *Mtb*, the lung, and should induce significant T cell responses in humans^12^,^13^,^22^.

To follow this track, we have designed a new algorithm to select the 50 most promising *Mtb* IVE-TB genes. We generated a new database by isolating *Mtb* RNA from the lung of highly susceptible (C3HeB/FeJ) as well as genetically resistant (C57BL/6J) mice both at early and late time points after aerosol infection, and analysing the expression of 2,068 *Mtb* genes to allow the selection of the most upregulated IVE-TB genes during early and late phase infection. By combining these data with analysis of high-level conservation in a set of 219 whole-genome sequenced *Mtb* complex strains, and with the presence of predicted HLA class Ia and II peptide binding motifs as well as high homology with other pathogenic mycobacteria, we could identify the most promising IVE-TB candidate antigens. Several of these were well recognized by blood cells from *in vitro Mtb* responsive donors and LTBI as measured by T-cell proliferation and multi-cytokine production assays, including antigens that did not induce IFN-γ. The fact that we identified several antigens which are currently in clinical vaccination trials independently underscores the validity of our novel *Mtb* antigen discovery approach as novel tools for TB vaccination and TB biomarker profiling.

The majority of the 50 IVE-TB genes selected in this study were found to be hyperconserved among all *Mtb* lineages as well as in pathogenic or non-tuberculous mycobacteria (NTM) and BCG. Although for other infectious agents^56^ antigen conservation is considered an essential feature to develop vaccines with global immunization coverage, this assumption is under debate in the TB field^57^. The finding that *Mtb* epitopes recognised by human T-cells are evolutionarily hyper-conserved has been interpreted to suggest that this may benefit the pathogen, and has promoted research into non-conserved, “hyper-variable” *Mtb* antigens and epitopes^58^. However, a possible, independent factor influencing this selection of hyper-conserved antigens might be that these *Mtb* sequences encode protein domains essentially required for *Mtb* survival inside the host cell, such that mutations could impair bacterial fitness and would be selected against^59^. Conversely, regions less essential to bacterial fitness might then allow more sequence variation, including epitope variation. *Mtb* indeed uses many other powerful immune evasion mechanisms than epitope variation, such as compromising antigen presentation by bacterial- directed vesicular antigen export^60^ and induction of immunoregulatory mechanisms to reduce CD4^+^ T cell recognition of *Mtb*-infected cells^61^. In addition, as others have suggested^62^, the observed epitope hyper-conservation might also be the result of previous BCG or NTM exposure, skewing *Mtb* recall antigen responses towards epitopes conserved among multiple mycobacterial species. Therefore, we consider important to include antigens with high homology with BCG/NTM given the opportunity to boost BCG/NTM-induced responses at a later stage in life. In any case, TB vaccination strategies will need to reprogram immune response profiles, including those against conserved antigens, such that protective effector mechanisms are activated without promoting pathology by imbalanced immune activation^59^. Therefore, we think that conserved IVE-TB antigens represent important targets for TB vaccination, including their ability to offer global *Mtb* lineage coverage.

The immunogenicity and immunodominance of the selected IVE-TB proteins were demonstrated by the fact that many of them stimulated significant lymphocyte proliferation and multiplex cytokine production in the blood of *Mtb* ESAT6/CFP10 (E/C)- and PPD- responsive *in vitro* individuals. Of note, the magnitude and the frequency of the antigen-induced responses were highly correlated. The use of independent assays and cohorts not only validated the results, but also revealed induction of several cytokines other than IFN-γ, considered relevant for further investigation of T cell responses.

Virtually all *Mtb* antigen discovery approaches thus far have relied on IFN-γ as single or as leading biomarker. Our study significantly broadens this to a wider range of cytokines. The recent failure of a TB vaccine candidate antigen identified mostly based on IFN-γ readouts^9^,^11^,^12^ urges the need for using novel correlates of immunogenicity^63^ at an early stage of TB vaccine development. Although some studies have analysed polyfunctional Th1-cells^16^,^63^,^64^ producing IFN-γ, TNF-α, and IL-2, their contribution in TB remains unclear^65^, although they seem to be associated primarily with vaccine induced protection in animal studies^66^. We have therefore included alternative cytokines which have been previously studied for their role in *Mtb* infection^67^,^68^,^69^,^70^. Interestingly, some IVE-TB antigens showed enhanced production of cytokines involved not only in Th1 but also in Th17 and Th2 responses. The ability of some IVE-TB antigens to induce Th2 related IL-13 response might be in line with our recent findings on *Mtb* specific HLA-E restricted CD8^+^ T-cells which had a ‘Th2-like’ profile, while still being able to actively lyse *Mtb* infected cells and inhibit intracellular *Mtb* outgrowth^67^. Future studies will address the cellular source(s) of the *Mtb* IVE-TB antigen induced alternative cytokines detected in this study. Of further note, distinct cytokine profiles (in terms of fold-change induction) were observed when selected IVE-TB antigens were compared to E/C. Most of these differences were more prominent for these alternative cytokines than for IFN-γ, underscoring the importance of characterising the immunogenic potential of novel antigens more broadly than by IFN-γ as a single biomarker.

The capability of the selected proteins to induce distinct cytokine responses was further validated in an independent cohort of LTBI donors. Indeed, a large group of IVE-TB antigens induced several cytokines and chemokine, especially TNF-α, IL-17, and IP-10, to an equal or even higher level than known *Mtb* antigens that were included for comparative purposes. As observed previously^28^, different Tuberculist classes of antigens elicited similar functional immune responses.

**Figure 8:**
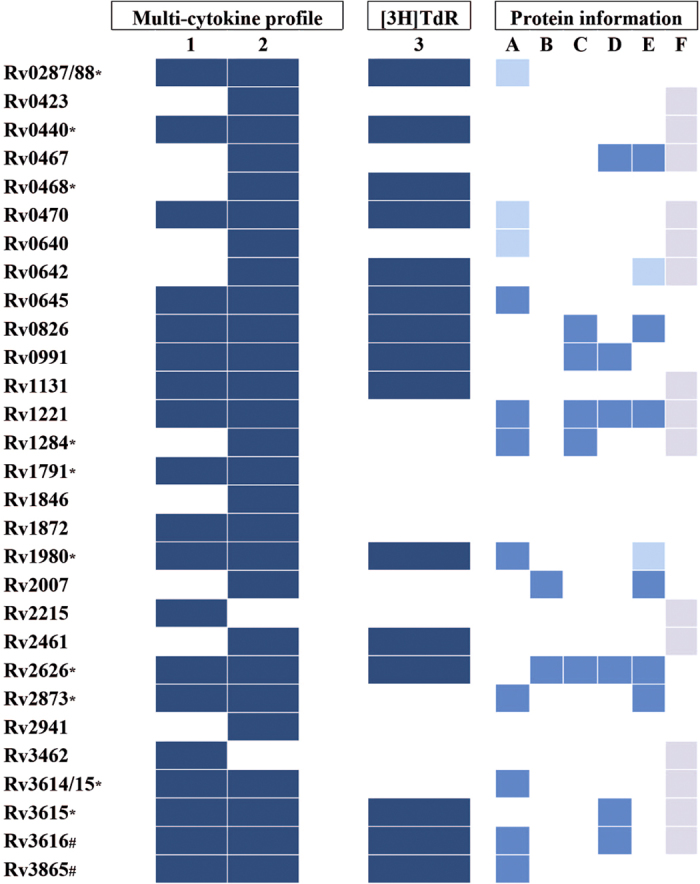
Multifunctional cytokine- and proliferative- responses induced by IVE-TB antigens, combined with literature-based functional assessment. Group of IVE-TB antigens which were able to induce multi-functional cytokine responses in at least one group of Mtb-exposed subjects as well as proliferative responses. 1: LTBI donors group (n = 25); 2: ESAT/CFP10 and PPD *in vitro*+ donors group (n = 9); 3: E/C and PPD *in vitro*+ donors group (n = 15);*: known antigen for IFN-ɣ production (41, 45, 46, 50); #: patent WO 2014063704 A2. A: Nutrient starvation 73; B: DosR 74; C: EHR 74; D: Vitamin C exposure 75; E: intra macrophage expressed genes 76; F: essential (according to Tuberculist). 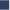: induction ≥2 cytokines; 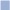: down-regulated; 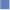: up-regulated; 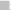: essential.

From the 48 tested IVE-TB proteins, a total of 29 were able to induce multi-functional cytokine responses in at least one group of *Mtb*-exposed subjects (Fig. 8). Fifteen out of 29 IVE-TB antigens also showed increased proliferation in terms of frequency and magnitude. The differences in cytokine profiles between the two groups of donors (likely exposed to NTM vs. *Mtb-*infected) might further be explained by the different assays used (PBMC vs. diluted whole blood) and donor to donor variability. The observed inter-donor variation in cytokine secretion, which agrees with other studies^71^, might also correspond to differences in the phenotype of responding cells, in the level and duration of *Mtb* exposure and in the genotype of the infecting *Mtb* strains^72^.

To the best of our knowledge, 17 out of these 29 IVE-TB antigens have not been described previously in terms of immunogenicity. Of note, three out of the 17 novel multifunctional IVE-TB antigens (Rv0645, Rv1131 and Rv2461) were among the seven proteins predicted to have the highest number of HLA class Ia and/or HLA class II peptide binding motifs, confirming the power of the *in-silico* prediction tools used in our discovery algorithm. Several of the 29 IVE-TB antigens identified here have been previously reported as being regulated in *in vitro* studies which aimed to replicate *in vivo* stress conditions that *Mtb* encounters inside host cells: the expression of some IVE-TB antigens was described to be affected by nutrient starvation^73^ (Rv0287/88, Rv0470c, Rv0640, Rv0645, Rv1221, Rv1284, Rv1980, Rv2873, Rv3614/15, Rv3616, Rv3865), hypoxia^74^ (Rv0826, Rv0991c, Rv1221, Rv1284, Rv2007, Rv2626c), exposure to vitamin C^75^ (Rv2626c, Rv0467, Rv1221, Rv0991, Rv3615c, Rv3616c) or intra-phagosomal infection in naïve and activated macrophages^76^ (Rv0467, Rv0642, Rv0826, Rv1121, Rv1980, Rv2007, Rv2626, Rv2873). This concordance is consistent with, and validates the contribution of some selected IVE-TB proteins in host-pathogen interactions in early and late phase *Mtb* infection and extends these results indirectly to *in vivo* pulmonary infection. In line with this, many of the IVE-TB antigens have been reported to be essential for *Mtb* (Fig. 8) and are highly homologous to proteins present in pathogenic or nontuberculous mycobacteria (NTM).

We conclude that the combination of our *in vivo* and *in silico* algorithm facilitates the identification of novel IVE-TB antigens, providing new tools for TB vaccination and antigen specific biomarker profiling. The high homology found with other pathogenic mycobacteria, such as *M. leprae* and *M. ulcerans*, might further extend their use as vaccine tools^77^,^78^. Finally, the generic approach we have presented here can be applied for targeted discovery of antigens to be used in control measures for other infectious disease.

## Methods

### Mouse strains and murine pulmonary infection for *Mtb* mRNA gene expression

C3HeB/FeJ (C3H) and C57BL/6J(B6) mice were purchased from The Jackson Laboratory (Bar Harbor, ME, USA) and housed under specific pathogen-free conditions. Mtb Erdman strain suspensions (Trudeau Institute, Saranac Lake, NY, USA) were grown to midlog phase, washed and stored at −80 °C. Before infection, mycobacteria were thawed, sonicated and diluted in PBS to 10^6^ CFU/ml. Mice were aerosol challenged by 25–50 CFU Mtb using a Madison chamber (University of Wisconsin, Madison, WI, USA)^79^. Four mice per group was sacrificed at five different time points. Studies in mice were performed in agreement with the Guide for the Care and Use of Laboratory Animals and the Animal Welfare Act. The study protocol (No. AN-15276.2016.05) was approved by the Institutional Animal Care and Use Committee of Boston University Medical Center.

### Genome-wide *Mtb* mRNA gene expression

Quantification of *Mtb* transcription profile was performed as previously described^19^,^23^,^80^,^81^. Total *Mtb* RNA was isolated from infected mouse lung tissue by homogenization in Trizol (Thermo Fisher Scientific, Waltham, MA, USA) and bacillary disruption by bead beating (MP Biomedicals, Solon, OH, USA). Total RNA was purified using RNeasy columns (Qiagen, Valencia, CA, USA). cDNA synthesis was conducted using 50ng total RNA, which was separated in reverse transcriptase (RT)+ and RT− reactions to control for DNA impurity. cDNA was further amplified via controlled multiplex pre-amplification^19^. Sequences and design of PCR primer/probe sets are available at http://genes.stanford.edu/technology.php and http://www.tbdb.org/rtpcrData.shtml. Individual gene transcript quantification was carried out using TaqMan primer/probe sets (Biosearch Technology, Petaluma, CA, USA). The cycle threshold values generated were transformed to relative gene copy numbers (RGCNs) based on logarithmic transformation/linear regression equations devised from calibration curves.

### Gene expression analysis

Based on genome-wide, real-time RT-PCR, *Mtb* gene expression patterns of 2,068 genes, mostly representing the first gene of each predicted operon, were analysed from the lungs of infected mice. Those included hypersusceptible C3HeB/FeJ mice^82^ and resistant C57BL/6J mice, after 2, 4, 6, 9 and 12 wks of *Mtb* challenge. For each time point, the median cycle threshold values of four mice per strain were converted to relative H37 Rv gene copy numbers (RGCNs). For each condition (time point and mouse strain) the RGCNs data were sorted from the highest to the lowest value; genes with RGCNs ranked in the top 15% (*n* = 310) of the ordered list generated 10 datasets (Fig. 1a). The threshold of 15% was chosen arbitrarily to limit the number of candidates for further analysis^23^. The expression levels observed for the first genes of each predicted operon are considered to extend to the co-regulated genes in the same operons. Microsoft Access 2010 was used to compare and retrieve data shared from all data sets (Table S1). Information on the functional classes was obtained from the Tuberculist database (http://tuberculist.epfl.ch/) (Fig. S1).

### Amino acid conservation

The level of conservation of the 194 candidate genes was determined in 219 whole-genome sequenced *Mtb* complex strains^83^, covering all seven known *Mtb* lineages^30^. The amino acid (aa) conservation of each gene was expressed as the ratio of number of aminoacid substitutions observed and the number of aa of the protein encoded by the examined gene. All the genes with a ratio of ≥98%, an arbitrarily chosen cut-off value, were considered hyper-conserved (Table S2).

### Structures of the proteins

For each gene, aa length and predicted transmembrane motifs were checked in the Tuberculist database (http://tuberculist.epfl.ch/) and Hidden Markov Models (http://www.cbs.dtu.dk/services/TMHMM/TMHMM2.0b.guide.php)^84^. Encoded proteins with a length >600 aa or including predicted transmembrane regions, with a probability above one or with multiple transmembrane regions, were excluded.

### Presence of peptide binding motifs for HLA class Ia and class II

The following methods were used with FASTA format input files: NetMHCcons 1.0 (cbs.dtu.dk/services/NetMHCcons) was employed to identify 9-mer peptides containing a peptide binding motif for 12 HLA class Ia supertype representatives (*HLA-A1, -A2, -A3*, -*A24, -A26, -B7, -B8, -B27, -B39, -B44, -B58* and *-B62*)^31^ and NetMHCIIpan 3.0 (cbs.dtu.dk/services/NetMHCIIpan)^32^ was employed to identify 15-mer peptides containing an HLA- peptide binding motif for 29 common HLA-DR, -DQ and -DP molecules, including 27 alleles: 15 HLA-DR, 6 HLA-DQ and 6 HLA-DP (DRB1*01:01; DRB1*03:01; DRB1*04:01; DRB1*07:01; DRB1*04:05; DRB1*08:02; DRB1*09:01; DRB1*11:01; DRB1*12:01; DRB1*13:02; DRB1*15:01; DRB3*01:01, DRB3*02:02; DRB4*01:01; DRB5*01:01;HLA DQA1*05:01-DQB1*02:01; HLA-DQA1*05:01-DQB1*03:01; HLA-DQA1*03:01-DQB1*03:02; HLA-DQA1*04:01-DQB1*04:02; HLA-DQA1*01:01-DQB1*05:01; HLA-DQA1*01:02-DQB1*06:02; HLA-DPA1*02:01-DPB1*01:01; HLA-DPA1*01:03-DPB1*02:01; HLA-DPA1*01:03-DPB1*04:01; HLA-DPA1*03:01-DPB1*04:02; HLA-DPA1*02:01-DPB1*05:01; HLA-DPA1*01:03-DPB1*14:01). These alleles represent approximately 50–75% of all allelic variants at each of the class II loci considered in the study of Greenbaum^34^, whereas DRB1_0803 and DRB1_1402 were extrapolated from a study on the functional similarities between the MHC-II molecules^85^. To quantitatively analyse the distribution of HLA alleles, we counted and summed the number of HLA class Ia alleles (including 12 HLA class Ia supertypes)^33^ and HLA class II alleles (including 17 HLA class II frequent alleles)^34^ covered by predicted peptide binding motifs for each protein of interest. If the sum was greater than 20, the protein was considered to have wide HLA coverage (Table S4). To avoid bias due to the protein size, the ratio of the number of predicted epitopes (NB) to the length of the proteins (len) was calculated (NB:len). The ranking of proteins with the highest number of predicted binding motifs was based on this ratio (Table S4).

### Protein sequence homology

The homology of selected proteins was studied using BLASTp program (http://blast.ncbi.nlm.nih.gov/Blast.cgi?PAGE=Proteins; January 2015). The query included BCG (taxid: 33892), *M. leprae* (taxid: 1769), *M. avium* (taxid: 1764), *M. ulcerans* (taxid: 1809) and *M. kansasii* (taxid: 1768). (Table S3).

### Recombinant proteins

As described previously^86^, *Mtb* genes were amplified by PCR from genomic H37Rv DNA and cloned by Gateway technology (Invitrogen, Carlsbad, CA, USA) in a bacterial expression vector containing a histidine (His) tag at the N-terminus. Vectors were overexpressed in *Escherichia coli (E. coli*) BL21 (DE3) and purified. The size and purity of the recombinant proteins were analysed by gel electrophoresis and western blotting with an anti-His Ab (Invitrogen) and an anti-*E. coli* polyclonal Ab (gift of Statens Serum Institute, Copenhagen, Denmark). Rv0287-Rv0288, Rv2346c-Rv2347, and Rv3614-Rv3615 were prepared as fusion proteins^29^ to mimic the pairwise dependent secretion pathway followed by T7S systems^87^. At this point, two proteins (Rv1197 and Rv1831) were excluded due to problems linked to their production. Forty-eight recombinant proteins were produced and tested to exclude protein-nonspecific T-cell stimulation and cellular toxicity^50^.

### Study subjects included

To assess T-cell proliferation after stimulation with the selected IVE-TB proteins, we used PBMC from 19 healthy Dutch donors. From these stimulated samples, the supernatant of 12 donors was tested with a multiplex cytokine assay (7-plex). Twenty-five latently *Mtb-*infected (LTBI) individuals, defined by a Mantoux tuberculin skin test (TST) ≥15 mm and/or a QuantiFERON-TB Gold In-Tube test (QFT-GIT) (Cellestis, Carnegie, VIC, Australia) ≥0.3 IU/ml, were recruited via the Dutch health service and diluted whole blood assay and multiplex cytokine array were performed. The study protocol (P07.048) was approved by the Institutional Board of the Leiden University Medical Center, The Netherlands. Informed written consent was required to participate in the study and was obtained before to collect blood samples. All experiments were performed in accordance with relevant guidelines and regulations.

### Lymphocyte stimulation test and thymidine incorporation assay

PBMC (1.5 × 10^5^) were cultured in AIM-V medium (Invitrogen, Breda, The Netherlands) in triplicate in 96-well round-bottom plates (Nunc, Roskilde, Denmark) and incubated at 37 °C and 5% CO_2._ The IVE-TB proteins were tested at a final concentration of 10 μg/ml. As controls, 5-μg/ml PPD (Statens Serum Institut, Copenhagen, Denmark) and 2-μg/ml PHA (Remel, Oxoid, Haarlem, The Netherlands) were included. After six days, the supernatants were used for cytokine determination as previously described^24^. Tritium thymidine was added for the last 16 hours of culture after which the cells were harvested and counted on a Microbetaplate counter (Wallac, Turku, Finland). The Stimulation Index (SI) was calculated as the ratio of the median counts per minute (cpm) of the stimulated samples to the median cpm without stimulation. SI values ≥ three were considered positive for proliferation.

### Multiplex cytokine array performed with PBMC supernatants

The concentrations of seven analytes (IL-13, IL-10, IL-17A, IFN-γ, IFN-γ–induced protein 10 [IP-10 (CXCL10)], GM-CSF and TNF-α) in the supernatants of stimulated PBMCs were assessed. Data were acquired using the Bio-Plex suspension array system powered by Luminex xMap multiplex technology (Bio-Rad Laboratories, Veenendaal, The Netherlands) and analysed using Bio-Plex Manager software 6.0 (Bio-Rad Laboratories), as previously described^88^. For all cytokines the background value of unstimulated samples was below 25 pg/ml and therefore we considered values >50 pg/ml as relevant. Only for IFN-γ, the background ranged between 23 and 83 pg/ml and therefore the cut-off was set at 100 pg/ml (in agreement with our previous studies). For IP-10, due to the large spread of the values measured in the unstimulated samples (37–8213 pg/ml), we subtracted the backgrounds, and we considered positive the responses over or equal than 250 pg/ml (three times the lowest detection limit of the IP-10 standard curve).

### Diluted whole blood assay

Within 3 hours of collection, heparinized venous blood was diluted 1:10 in AIM-V medium (Invitrogen, Breda, The Netherlands). Samples were incubated (450 μl/well) in 48-well plates at 37 °C at 5% CO_2_, 90% relative humidity, with 50 μl antigen solution (final concentration of 10 μg/ml). After 24 hours, 200 μl of the supernatants were collected from each well and frozen in aliquots at −20 °C until further analysis.

### Multiple cytokine array and analysis of diluted whole blood supernatant

A human R&D^TM^ premixed Multi-analyte kit was used to measure IL-13, IL-22, IL-17A, IFN-γ, IP-10, IL-9 and TNF-α in diluted whole blood culture supernatants according to manufacturer’s instructions. Values outside the upper or lower limits of quantification were set as the values of the analyte detection limits.

### Statistical analysis

Statistical analysis was performed using Graph Pad Prism (version 6.0). A Spearman nonparametric correlation was calculated to compare the immunodominance and immunogenicity of the IVE-TB antigens in different immunoassays. The ranking response frequency was calculated as follows: for each antigen the number of positive hits observed in the group of donors analysed was counted and the sum of positive responses for each antigen was obtained. These sums were used to rank the antigens according to their ability to induce a positive response. Similarly, the ranking response magnitude was based on the cumulative response measured among donors to each antigen^55^. The Mann–Whitney test was used to compare the difference between groups of donors based on the number of antigens recognised and the magnitude of response induced by stimulation with the antigens. Dunn’s multiple comparisons test was performed to analyse the difference between each antigen and the unstimulated samples for all cytokines measured in the LTBI samples. A p- value less than 0.05 was considered significant.

## Additional Information

**How to cite this article**: Coppola, M. *et al*. New Genome-Wide Algorithm Identifies Novel *In-Vivo* Expressed *Mycobacterium Tuberculosis* Antigens Inducing Human T-Cell Responses with Classical and Unconventional Cytokine Profiles. *Sci. Rep.* **6**, 37793; doi: 10.1038/srep37793 (2016).

**Publisher's note:** Springer Nature remains neutral with regard to jurisdictional claims in published maps and institutional affiliations.

## Supplementary Material

Supplementary Information
