# Supplementary material for: New Genome-Wide Algorithm Identifies Novel In-Vivo Expressed Mycobacterium Tuberculosis Antigens Inducing Human T-Cell Responses with Classical and Unconventional Cytokine Profiles
Source: Sci Rep. 2016 Nov 28;6:37793. doi: 10.1038/srep37793 (PMC5125271; doi:10.1038/srep37793)
Supplement: Supplementary Information [file srep37793-s1.pdf]

Supplementary Information

New Genome-Wide Algorithm Identifies Novel *In-Vivo* Expressed *Mycobacterium Tuberculosis* Antigens Inducing Human T-Cell Responses with Classical and Unconventional Cytokine Profiles.

Mariateresa Coppola[1], Krista E van Meijgaarden[1], Kees LMC Franken[1], Susanna Commandeur[1,#a], Gregory Dolganov[2], Igor Kramnik[3], Gary K Schoolnik[2], Inaki Comas[4,5], Ole Lund[6], Corine Prins[1], Susan JF van den Eeden[1], Gro E Korsvold[7], Fredrik Oftung[7], Annemieke Geluk[1¶] and Tom HM Ottenhoff [1¶*]

[1] Department of Infectious Diseases, Leiden University Medical Center, Leiden, The Netherlands; [2] Department Microbiology Immunology, Stanford Univ. School of Medicine, Stanford, USA;[3] Department Immunology Infectious Diseases, Harvard School of Public Health, Boston, USA;[4]Institute of Biomedicine of Valencia (IBV-CSIC) Valencia, Spain; [5] CIBER in Epidemiology and Public Health, Madrid, Spain; [6] Dept. Systems Biology, Technical Univ. Denmark;[7] Department of Infectious Disease Immunology, Domain for Infection Control and Environmental Health, Norwegian Institute of Public Health, Oslo, Norway.

[#a]: Current Address: Dept. of Medical Microbiology and Infection Control, VU University Medical Center, Amsterdam, The Netherlands

[¶]: These authors are joint senior authors.

* T.H.M.Ottenhoff@lumc.nl

Supplemental Figure Legends

Figure S1. Comparison of the functional classes of the top 15% up-regulated IVE-TB genes compared to the whole *Mtb* genome. The pie-charts (A) indicate the proportion of each functional category (as indicated in Tuberculist) of the top 15% highly *in vivo* expressed *Mtb* (IVE-TB) genes (*n* = 194) and of the whole *Mtb* genome (*n* = 4111). The histogram (B) shows the ratio of the two proportions; ratios less or greater than 1 suggest the under- or over-representation of the indicated functional class in the top 15% IVE-TB genes, respectively.

Figure S2. Immunodominance and immunogenicity of IVE-TB antigens based on cytokine production. PBMCs of *Mtb*-responders (*n* = 9) were stimulated with IVE-TB antigens and tested for the level of multiple cytokines by multiplex assay. The frequency (x-axis) and the magnitude (y-axis) of responses were ranked to compare the immunodominance and the immunogenicity for each antigen per analyte as well as for positive controls (PPD and PHA). In quadrant A are the top antigens for immunogenicity, in quadrant C the top antigens for immunodominance, and in quadrant B the top antigens for both parameters. Results were compared by Spearman's rank correlation coefficient. Graphs are shown for IL-13and GM-CSF.

Figure S3. Functional classes of IVE-TB antigens recognised by latently *Mtb*-infected (LTBI) individuals. Functional categories (as described in Tuberculist) are displayed for the IVE-TB antigens able to induce (A) four, (B) three, (C) two or (D) one cytokines in LTBI donors. The functional category of the *Mtb* protein does not correlate with the type of responses observed.

Figure S1

A

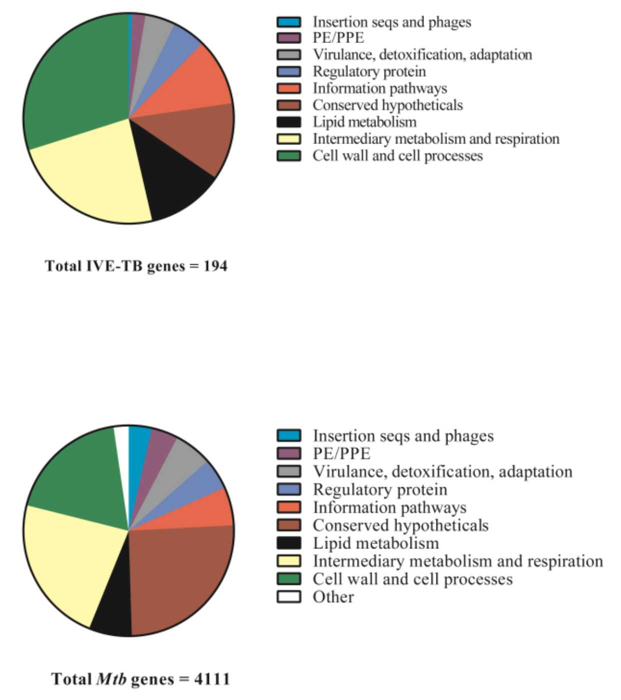
Insertion seqs and phages
PE/PPE
Virulance, detoxification, adaptation
Regulatory protein
Information pathways
Conserved hypotheticals
Lipid metabolism
Intermediary metabolism and respiration
Cell wall and cell processes
Total IVE-TB genes = 194
Insertion seqs and phages
PE/PPE
Virulance, detoxification, adaptation
Regulatory protein
Information pathways
Conserved hypotheticals
Lipid metabolism
Intermediary metabolism and respiration
Cell wall and cell processes
Other
Total Mtb genes = 4111

B

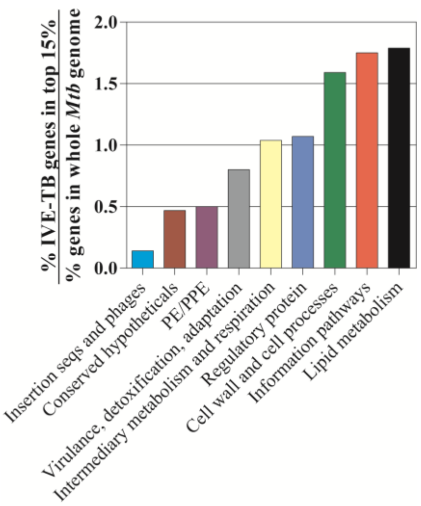
% IVE-TB genes in top 15%
% genes in whole Mtb genome
2.0
1.5
1.0
0.5
0.0
Insertion seqs and phages
Conserved hypotheticals
PE/PPE
Virulance, detoxification, adaptation
Intermediary metabolism and respiration
Regulatory protein
Cell wall and cell processes
Information pathways
Lipid metabolism

Figure S2

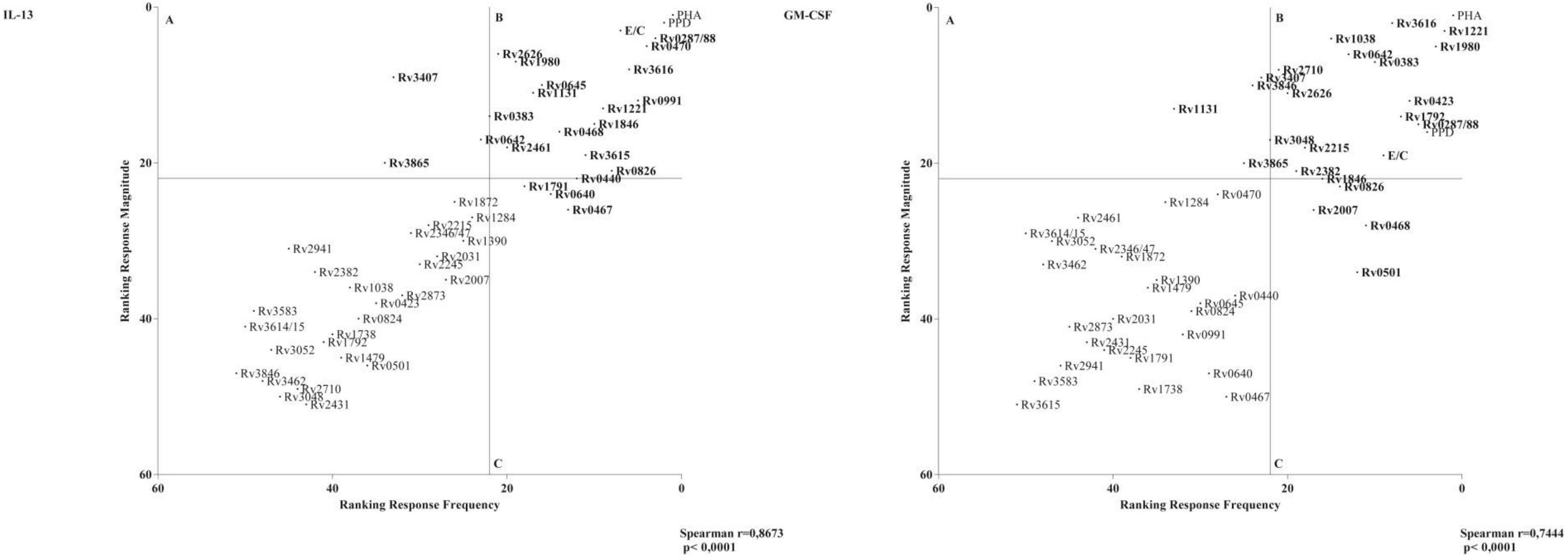
IL-13
A
B
C
Ranking Response Magnitude
Ranking Response Frequency
0
20
40
60
PHA
PPD
E/C
Rv0287/88
Rv0470
Rv2626
Rv1980
Rv3616
Rv3407
Rv0645
Rv1131
Rv0991
Rv1221
Rv0383
Rv1846
Rv0468
Rv0642
Rv2461
Rv3615
Rv3865
Rv0826
Rv0440
Rv1791
Rv0640
Rv0467
Rv1872
Rv1284
Rv2215
Rv2346/47
Rv1390
Rv2941
Rv2031
Rv2245
Rv2382
Rv2007
Rv1038
Rv2873
Rv0423
Rv3583
Rv0824
Rv3614/15
Rv1738
Rv1792
Rv3052
Rv1479
Rv0501
Rv3846
Rv3462
Rv2710
Rv3048
Rv2431
Spearman r=0,8673
p< 0,0001
GM-CSF
A
B
C
Ranking Response Magnitude
Ranking Response Frequency
0
20
40
60
PHA
Rv3616
Rv1221
Rv1038
Rv1980
Rv0642
Rv0383
Rv2710
Rv3407
Rv3846
Rv2626
Rv0423
Rv1131
Rv1792
Rv0287/88
PPD
Rv3048
Rv2215
E/C
Rv3865
Rv2382
Rv1846
Rv0826
Rv0470
Rv1284
Rv2007
Rv2461
Rv0468
Rv3614/15
Rv3052
Rv2346/47
Rv1872
Rv3462
Rv0501
Rv1390
Rv1479
Rv0440
Rv0645
Rv0824
Rv2031
Rv2873
Rv0991
Rv2431
Rv2245
Rv1791
Rv2941
Rv0640
Rv3583
Rv1738
Rv0467
Rv3615
Spearman r=0,7444
p< 0,0001

Figure S3

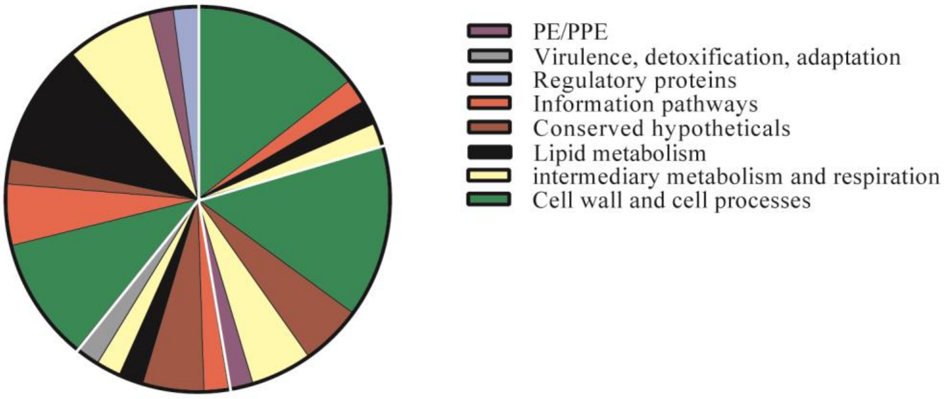

PE/PPE
Virulence, detoxification, adaptation
Regulatory proteins
Information pathways
Conserved hypotheticals
Lipid metabolism
intermediary metabolism and respiration
Cell wall and cell processes

Table S1. *List of the 194 highly expressed in–vivo Mtb (IVE-TB) genes identified in the lung of Mtb infected mice independent of their genetic susceptibility to tuberculosis.*

H37Rv identity	Described (predicted) function
Rv0046c	Myo-inositol-1-phosphate synthase Ino1
Rv0065	Possible toxin VapC1
Rv0066c	Probable isocitrate dehydrogenase [NADP] Icd2 (oxalosuccinate decarboxylase)
Rv0103c	Probable cation-transporter P-type ATPase B CtpB
Rv0140	Conserved protein
Rv0147	Probable aldehyde dehydrogenase (NAD+) dependent
Rv0188	Probable conserved transmembrane protein
Rv0206c	Possible conserved transmembrane transport protein MmpL3
Rv0211	iron-regulated phosphoenolpyruvate carboxykinase pckA
Rv0244c	Probable acyl-CoA dehydrogenase FadE5
Rv0249c	Probable succinate dehydrogenase
Rv0284	ESX conserved component EccC3.
Rv0287	Esat-6 like protein esxG
Rv0288	Low molecular weight protein antigen 7 esxH
Rv0383c	Possible conserved secreted protein
Rv0423c	Probable thiamine biosynthesis protein ThiC
Rv0440	60 kDa chaperonin 2 GroEL2
Rv0451c	Probable conserved membrane protein MmpS4
Rv0453	PPE family protein PPE11
Rv0467	Isocitrate lyase Icl
Rv0468	3-hydroxybutyryl-CoA dehydrogenase FadB2
Rv0469	Possible mycolic acid synthase UmaA
Rv0470c	Mycolic acid synthase PcaA
Rv0474	Probable transcriptional regulatory protein
Rv0476	Possible conserved transmembrane protein
Rv0501	Possible UDP-glucose 4-epimerase GalE2
Rv0572c	Hypothetical protein
Rv0638	Probable preprotein translocase SecE1
Rv0640	50S ribosomal protein L11 RplK
Rv0642c	Methoxy mycolic acid synthase 4 MmaA4
Rv0645c	Methoxy mycolic acid synthase 1 MmaA1
Rv0711	Possible arylsulfatase AtsA
Rv0715	50S ribosomal protein L24 RplX
Rv0717	30S ribosomal protein S14 RpsN1
Rv0759c	Conserved hypothetical protein
Rv0761c	Possible zinc-containing alcohol dehydrogenase NAD dependent AdhB
Rv0805	Class III cyclic nucleotide phosphodiesterase
Rv0815c	Probable thiosulfate sulfurtransferase CysA2
Rv0824c	Probable acyl-[acyl-carrier protein] desaturase DesA1
Rv0826	Conserved hypothetical protein
Rv0884c	Possible phosphoserine aminotransferase SerC
Rv0932c	Periplasmic phosphate-binding lipoprotein PstS2
Rv0951	Probable succinyl-CoA synthetase
Rv0986	Probable adhesion component transport ATP-binding protein ABC transporter
Rv0991	Conserved serine rich protein
Rv1013	Putative polyketide synthase Pks16
Rv1037c	Putative ESAT-6 like protein EsxI
Rv1038c	Esat-6 like protein esxJ
Rv1093	Serine hydroxymethyltransferase 1 GlyA1
Rv1109c	Conserved protein
Rv1131	Probable methylcitrate synthase PrpC
Rv1156	Conserved protein
Rv1174c	Low molecular weight T-cell antigen TB8.4
Rv1182	Probable conserved polyketide synthase associated protein PapA3

Rv1194c	Conserved protein
Rv1197	ESAT-6 like protein EsxK
Rv1221	Alternative RNA polymerase sigma factor sigE
Rv1248c	Multifunctional alpha-ketoglutarate metabolic enzyme
Rv1270c	Possible lipoprotein LprA
Rv1274	Possible lipoprotein LprB
Rv1284	Beta-carbonic anhydrase
Rv1286	Probable bifunctional enzyme CysN/CysC
Rv1297	Probable transcription termination factor Rho homolog
Rv1298	50S ribosomal protein L31 RpmE
Rv1303	Conserved hypothetical transmembrane protein
Rv1304	Probable ATP synthase a chain AtpB
Rv1305	Probable ATP synthase C chain AtpE
Rv1308	Probable ATP synthase alpha chain AtpA
Rv1324	Possible thioredoxin
Rv1331	Conserved hypothetical protein
Rv1335	Sulfur carrier protein CysO
Rv1388	Putative integration host factor MihF
Rv1390	Probable DNA-directed RNA polymerase
Rv1398	Possible antitoxin VapB10
Rv1404	Probable transcriptional regulatory protein
Rv1411c	Conserved lipoprotein LprG
Rv1419	Unknown protein
Rv1440	Probable protein-export membrane protein
Rv1449c	Transketolase Tkt
Rv1467c	Probable acyl-CoA dehydrogenase FadE15
Rv1471	Probable thioredoxin TrxB1
Rv1479	Probable transcriptional regulatory protein MoxR1
Rv1611	Probable indole-3-glycerol phosphate synthase TrpC
Rv1630	30S ribosomal protein S1 RpsA
Rv1636	Iron-regulated universal stress protein family protein TB15.3
Rv1641	Probable initiation factor if-3 InfC
Rv1738	Conserved hypothetical protein
Rv1783	ESX conserved component EccC5
Rv1784	Conserved hypothetical protein
Rv1791	PE family protein PE19
Rv1792	ESAT-6 like protein EsxM
Rv1793	Putative ESAT-6 like protein EsxN
Rv1794	Conserved protein
Rv1795	ESX conserved component EccD5
Rv1805c	Hypothetical protein
Rv1829	Conserved protein
Rv1831	Hypothetical protein
Rv1846c	Transcriptional repressor BlaI
Rv1854c	Probable NADH dehydrogenase Ndh
Rv1872c	Possible L-lactate dehydrogenase
Rv1876	Probable bacterioferritin BfrA
Rv1880c	Probable cytochrome P450 140 Cyp140
Rv1888c	Possible transmembrane protein
Rv1909c	Ferric uptake regulation protein FurA
Rv1915	Probable isocitrate lyase AceAa
Rv1925	Probable acyl-CoA ligase FadD31
Rv1980c	Immunogenic protein Mpt64
Rv1981c	Ribonucleoside-diphosphate reductase
Rv1984c	Probable cutinase precursor CFP21
Rv2007	Ferredoxin fdxA
Rv2031	Heat shock protein hspX
Rv2091c	Probable membrane protein
Rv2108	PPE family protein PPE36
Rv2115c	Mycobacterial proteasome ATPase Mpa

Rv2193	Probable cytochrome C oxidase (subunit III) CtaE
Rv2194	Probable ubiquinol-cytochrome C reductase QcrC
Rv2215	DlaT, dihydrolipoamide acyltransferase, E2 component of pyruvate dehydrogenase
Rv2219	Probable conserved transmembrane protein
Rv2245	3-oxoacyl-[acyl-carrier protein] synthase 1 KasA
Rv2334	Cysteine synthase a CysK1
Rv2346c	Putative ESAT-6 like protein EsxO
Rv2347c	Putative ESAT-6 like protein EsxP
Rv2382	Polyketide synthetase mbtC
Rv2389c	Probable resuscitation-promoting factor RpfD
Rv2428	Alkyl hydroperoxide reductase C protein AhpC
Rv2431c	PE family protein PE25
Rv2455c	Probable oxidoreductase
Rv2457c	Probable ATP-dependent CLP protease ATP-binding subunit ClpX
Rv2460c	Probable ATP-dependent CLP protease proteolytic subunit 2 ClpP2
Rv2461c	Probable ATP-dependent CLP protease proteolytic subunit 1 ClpP1
Rv2582	Probable peptidyl-prolyl cis-trans isomerase B PpiB
Rv2617c	Probable transmembrane protein
Rv2626	Hypoxic response protein 1 Hrp1
Rv2657c	Probable PhiRv2 prophage protein
Rv2663	Hypothetical protein
Rv2699c	Conserved hypothetical protein
Rv2710	RNA polymerase sigma factor sigB
Rv2716	Conserved protein
Rv2873	Cell surface lipoprotein Mpt83
Rv2875	Major secreted immunogenic protein Mpt70
Rv2876	Possible conserved transmembrane protein
Rv2878c	Soluble secreted antigen Mpt53 precursor
Rv2882c	Ribosome recycling factor Frr
Rv2890c	30S ribosomal protein S2 RpsB
Rv2931	Phenolpthiocerol synthesis type-I polyketide synthase PpsA
Rv2932	Phenolpthiocerol synthesis type-I polyketide synthase PpsB
Rv2934	Phenolpthiocerol synthesis type-I polyketide synthase PpsD
Rv2935	Phenolpthiocerol synthesis type-I polyketide synthase PpsE
Rv2936	Daunorubicin-dim-transport ATP-binding protein ABC transporter DrrA
Rv2939	Possible conserved polyketide synthase associated protein PapA5
Rv2941	Fatty-acid-AMP ligase FadD28
Rv3048c	Ribonucleoside-diphosphate reductase
Rv3051c	Ribonucleoside-diphosphate reductase NrdE (R1F protein)
Rv3052	hypothetical protein nrdI
Rv3141	Probable NADPH quinone oxidoreductase FadB4
Rv3193c	Probable conserved transmembrane protein
Rv3213c	Possible SOJ/para-related protein
Rv3219	Transcriptional regulator whib-like whiB1
Rv3246c	Two component sensory transduction transcriptional regulatory protein MtrA
Rv3248c	Probable adenosylhomocysteinase SahH
Rv3280	Probable propionyl-CoA carboxylase beta chain 5 AccD5
Rv3407	Possible antitoxin VapB47
Rv3416	Transcriptional regulatory protein WhiB-like WhiB3
Rv3418c	10 kDa chaperonin GroES
Rv3460c	30S ribosomal protein S13 RpsM
Rv3462	Translation initiation factor IF-1 infA
Rv3528c	Unknown protein
Rv3583c	Possible transcription factor
Rv3596c	Probable ATP-dependent protease ATP-binding subunit ClpC1
Rv3601c	Probable aspartate 1-decarboxylase precursor PanD
Rv3614c	ESX-1 secretion-associated protein EspD
Rv3615	ESX-1 secretion-associated protein EspC
Rv3616	Conserved alanine and glycine rich protein
Rv3619c	Putative ESAT-6 like protein EsxV

Rv3620c	Putative ESAT-6 like protein EsxW
Rv3667	Acetyl-coenzyme A synthetase Acs
Rv3681c	Probable transcriptional regulatory protein WhiB-like WhiB4
Rv3682	Probable bifunctional membrane-associated penicillin-binding protein 1A/1B PonA2
Rv3824c	Conserved polyketide synthase associated protein PapA1
Rv3841	Bacterioferritin BfrB
Rv3846	Superoxide dismutase sodA
Rv3854c	Monooxygenase EthA
Rv3857c	Possible membrane protein
Rv3858c	Probable NADH-dependent glutamate synthase
Rv3863	Unknown alanine rich protein
Rv3864	ESX-1 secretion-associated protein EspE
Rv3865	ESX-1 secretion-associated protein EspF
Rv3874	10 kDa culture filtrate antigen EsxB (LHP) (CFP10)
Rv3875	6 kda early secretory antigenic target esxA
Rv3878	ESX-1 secretion-associated protein EspJ
Rv3879c	ESX-1 secretion-associated protein EspK
Rv3881c	Secreted ESX-1 substrate protein B, EspB
Rv3890c	ESAT-6 like protein EsxC
Rv3914	Thioredoxin trxC

Table S2. *Conservation and number of predicted HLA class-Ia and -II binding peptides of 194 IVE-TB genes.*

Genes	Aminoacid (aa) change		Ratio N/S	Protein length	Aa conservation (%)	NB HLA class Ia	NB HLA class II
	Synonymous (S)	Nonsynonymous (N)					
Rv0046c	3	5	1,67	367	99	68	2270
Rv0065	2	3	1,50	133	98	45	998
Rv0066c	7	12	1,71	745	98	186	4940
Rv0103c	8	11	1,38	752	99	205	5666
Rv0140	1	4	4,00	126	97	38	579
Rv0147	5	12	2,40	506	98	140	3800
Rv0188	5	0	0,00	143	100	57	1213
Rv0206c	11	17	1,55	944	98	292	7097
Rv0211	7	11	1,57	606	98	133	2869
Rv0244c	6	17	2,83	611	97	148	4594
Rv0249c	3	3	1,00	273	99	104	2810
Rv0284	17	17	1,00	1330	99	347	9270
Rv0287	0	1	NA	97	99	25	813
Rv0288	1	6	6,00	96	94	26	609
Rv0383c	2	3	1,50	284	99	62	1613
Rv0423c	4	4	1,00	547	99	118	3113
Rv0440	12	0	0,00	540	100	92	2733
Rv0451c	3	1	0,33	140	99	34	511
Rv0453	0	0	NA	518	100	135	3249
Rv0467	7	1	0,14	428	100	100	2399
Rv0468	4	7	1,75	286	98	60	2055
Rv0469	3	10	3,33	286	97	84	2519
Rv0470c	2	3	1,50	287	99	76	2252
Rv0474	5	2	0,40	140	99	20	821
Rv0476	0	0	NA	87	100	33	717
Rv0501	2	6	3,00	376	98	88	2816
Rv0572c	1	4	4,00	113	96	23	522
Rv0638	0	2	NA	161	99	43	888
Rv0640	1	1	1,00	142	99	22	782
Rv0642c	5	4	0,80	301	99	71	1805
Rv0645c	5	2	0,40	286	99	80	2479
Rv0711	9	10	1,11	787	99	177	4322
Rv0715	3	0	0,00	105	100	12	348
Rv0717	0	2	NA	61	97	13	394
Rv0759c	1	1	1,00	110	99	31	807
Rv0761c	2	2	1,00	375	99	71	1961
Rv0805	3	7	2,33	318	98	66	2057
Rv0815c	2	0	0,00	277	100	49	1238
Rv0824c	3	3	1,00	338	99	77	2160
Rv0826	1	7	7,00	351	98	89	2172
Rv0884c	3	4	1,33	376	99	94	2195
Rv0932c	4	9	2,25	370	98	68	1481
Rv0951	3	5	1,67	387	99	72	2305
Rv0986	2	4	2,00	248	98	57	1533
Rv0991	1	0	0,00	110	100	14	387
Rv1013	2	0	0,00	544	100	136	3600
Rv1037c	1	0	0,00	94	100	11	475
Rv1038c	1	1	1,00	98	99	17	468
Rv1093	4	3	0,75	426	99	96	2870
Rv1109c	1	6	6,00	212	97	50	1445
Rv1131	6	7	1,17	393	98	121	2960
Rv1156	3	4	1,33	195	98	43	1288
Rv1174c	0	2	NA	110	98	20	708
Rv1182	5	4	0,80	472	99	118	2787
Rv1194c	3	8	2,67	421	98	96	3016
Rv1197	1	1	1,00	98	99	17	477
Rv1221	2	4	2,00	257	98	58	1546
Rv1248c	15	8	0,53	1231	99	261	8749
Rv1270c	3	5	1,67	244	98	36	1183
Rv1274	0	1	NA	185	99	36	745
Rv1284	4	1	0,25	163	99	32	1026
Rv1286	7	8	1,14	614	99	129	3980
Rv1297	2	7	3,50	602	99	95	2856
Rv1298	1	1	1,00	80	99	6	149
Rv1303	1	1	1,00	161	99	55	1599

Genes	Aminoacid (aa) change		Ratio N/S	Protein length	Aa conservation (%)	NB HLA class Ia	NB HLA class II
	Synonymous (S)	Nonsynonymous (N)					
**Rv1304**	3	3	1,00	250	99	114	2804
**Rv1305**	2	0	0,00	81	100	25	481
**Rv1308**	2	5	2,50	549	99	119	2799
**Rv1324**	3	4	1,33	304	99	65	1971
**Rv1331**	1	1	1,00	101	99	25	513
**Rv1335**	1	1	1,00	93	99	16	371
**Rv1388**	1	1	1,00	190	99	33	756
**Rv1390**	0	0	NA	110	100	30	546
**Rv1398**	1	1	1,00	85	99	9	418
**Rv1404**	3	3	1,00	160	98	39	1285
**Rv1411c**	2	1	0,50	236	100	36	1101
**Rv1419**	2	3	1,50	157	98	23	441
**Rv1440**	0	2	NA	77	97	22	414
**Rv1449c**	4	7	1,75	700	99	157	4241
**Rv1467c**	2	10	5,00	609	98	141	4331
**Rv1471**	0	0	NA	123	100	22	788
**Rv1479**	2	4	2,00	377	99	93	3078
**Rv1611**	1	3	3,00	272	99	53	1417
**Rv1630**	4	4	1,00	481	99	74	2076
**Rv1636**	1	3	3,00	146	98	20	689
**Rv1641**	2	2	1,00	201	99	24	1015
**Rv1738**	1	1	1,00	94	99	14	439
**Rv1783**	3	3	1,00	435	99	120	2812
**Rv1784**	13	11	0,85	932	99	226	6678
**Rv1791**	0	0	NA	99	100	28	761
**Rv1792**	0	0	NA	98	100	17	484
**Rv1793**	4	1	0,25	94	99	12	549
**Rv1794**	4	1	0,25	300	100	70	1766
**Rv1795**	3	4	1,33	503	99	179	4687
**Rv1805c**	1	2	2,00	115	98	25	401
**Rv1829**	3	2	0,67	164	99	31	1049
**Rv1831**	0	0	NA	85	100	12	446
**Rv1846c**	3	4	1,33	138	97	30	784
**Rv1854c**	7	5	0,71	463	99	110	3358
**Rv1872c**	5	14	2,80	414	97	98	3068
**Rv1876**	0	3	NA	159	98	30	1187
**Rv1880c**	8	7	0,88	438	98	93	3192
**Rv1888c**	2	2	1,00	186	99	116	1964
**Rv1909c**	0	5	NA	150	97	32	817
**Rv1915**	3	8	2,67	367	98	86	2688
**Rv1925**	3	6	2,00	620	99	149	4268
**Rv1980c**	3	2	0,67	228	99	43	1106
**Rv1981c**	1	1	1,00	322	100	90	2198
**Rv1984c**	1	2	2,00	217	99	40	970
**Rv2007**	0	1	NA	114	99	14	389
**Rv2031**	4	2	0,50	144	99	25	694
**Rv2091c**	5	2	0,40	244	99	32	477
**Rv2108**	0	0	NA	243	100	60	1549
**Rv2115c**	7	4	0,57	609	99	114	3396
**Rv2193**	1	2	2,00	203	99	123	2010
**Rv2194**	2	6	3,00	280	98	68	1165
**Rv2215**	5	10	2,00	553	98	96	2239
**Rv2219**	3	8	2,67	250	97	61	1548
**Rv2245**	4	7	1,75	416	98	93	2163
**Rv2334**	4	5	1,25	310	98	68	1839
**Rv2346c**	2	1	0,50	94	99	13	540
**Rv2347c**	1	0	0,00	98	100	19	479
**Rv2382**	5	1	0,20	444	100	89	2567
**Rv2389c**	3	0	0,00	154	100	20	625
**Rv2428**	5	1	0,20	195	99	37	972
**Rv2431c**	0	0	NA	99	100	31	807
**Rv2455c**	5	9	1,80	653	99	159	3863
**Rv2457c**	11	5	0,45	426	99	96	2618
**Rv2460c**	1	1	1,00	214	100	52	1681
**Rv2461c**	4	1	0,25	200	100	62	1624

Genes	Aminoacid (aa) change Synonymous (S)	Nonsynonymous (N)	Ratio N/S	Protein length	Aa conservation (%)	NB HLA class Ia	NB HLA class II
**Rv2582**	3	3	1,00	308	99	59	1482
**Rv2617c**	1	3	3,00	146	98	47	1358
**Rv2626**	1	2	2,00	143	99	36	787
**Rv2657c**	0	0	NA	86	100	22	524
**Rv2663**	0	2	NA	77	97	27	496
**Rv2699c**	0	2	NA	100	98	15	296
**Rv2710**	3	1	0,33	323	100	58	2109
**Rv2716**	1	2	2,00	228	99	42	1073
**Rv2873**	3	3	1,00	220	99	42	1102
**Rv2875**	2	3	1,50	193	98	42	1072
**Rv2876**	2	4	2,00	104	96	30	619
**Rv2878c**	1	4	4,00	173	98	51	1191
**Rv2882c**	4	4	1,00	185	98	27	916
**Rv2890c**	3	1	0,33	287	100	59	1680
**Rv2931**	15	25	1,67	1876	99	393	11518
**Rv2932**	16	7	0,44	1538	100	356	10367
**Rv2934**	8	23	2,88	1827	99	407	11483
**Rv2935**	13	20	1,54	1488	99	373	9629
**Rv2936**	2	6	3,00	331	98	78	2239
**Rv2939**	3	7	2,33	422	98	115	3127
**Rv2941**	11	6	0,55	580	99	135	3925
**Rv3048c**	3	0	0,00	324	100	105	2332
**Rv3051c**	4	6	1,50	693	99	188	5119
**Rv3052**	0	0	NA	150	100	40	874
**Rv3141**	2	5	2,50	323	98	64	1869
**Rv3193c**	13	12	0,92	992	99	282	7902
**Rv3213c**	4	7	1,75	266	97	61	1907
**Rv3219**	1	0	0,00	84	100	12	150
**Rv3246c**	1	2	2,00	228	99	47	1480
**Rv3248c**	8	7	0,88	495	99	87	2728
**Rv3280**	6	4	0,67	548	99	119	3349
**Rv3407**	0	1	NA	99	99	22	534
**Rv3416**	0	3	NA	102	97	24	214
**Rv3418c**	1	0	0,00	100	100	16	279
**Rv3460c**	2	2	1,00	124	98	22	519
**Rv3462**	3	0	0,00	73	100	16	556
**Rv3528c**	4	4	1,00	237	98	53	1555
**Rv3583c**	0	0	NA	162	100	30	800
**Rv3596c**	9	6	0,67	848	99	184	5124
**Rv3601c**	3	1	0,33	139	99	22	750
**Rv3614c**	3	5	1,67	184	97	42	860
**Rv3615**	2	2	1,00	103	98	15	543
**Rv3616**	4	9	2,25	392	98	82	2583
**Rv3619c**	0	0	NA	94	100	11	475
**Rv3620c**	0	2	NA	98	98	17	468
**Rv3667**	7	13	1,86	651	98	152	3698
**Rv3681c**	1	5	5,00	118	96	28	457
**Rv3682**	10	8	0,80	810	99	165	4239
**Rv3824c**	9	9	1,00	511	98	122	3436
**Rv3841**	3	3	1,00	181	98	46	1297
**Rv3846**	3	4	1,33	207	98	52	1581
**Rv3854c**	10	11	1,10	489	98	138	3412
**Rv3857c**	1	0	0,00	65	100	13	366
**Rv3858c**	7	3	0,43	488	99	88	1965
**Rv3863**	5	11	2,20	392	97	62	2023
**Rv3864**	2	5	2,50	402	99	78	2436
**Rv3865**	0	3	NA	103	97	22	558
**Rv3874**	0	2	NA	100	98	11	288
**Rv3875**	2	1	0,50	95	99	8	341
**Rv3878**	0	7	NA	280	98	34	963
**Rv3879c**	12	17	1,42	729	98	133	2916
**Rv3881c**	4	19	4,75	460	96	81	1493
**Rv3890c**	1	0	0,00	95	100	16	377
**Rv3914**	1	4	4,00	116	97	30	601

Aa changes were determined in 219 whole-genome sequenced Mtb complex strains[83]; NB: number of peptides predicted to bind to 12 HLA class Ia supertype representatives[31] and to 29 HLA class II molecules[34]. Binding peptides are identified based on % Rank as described in cbs.dtu.dk/services/NetMHCcons[31] and cbs.dtu.dk/services/NetMHCIIpan[32]servers.

Table S3. *Aminoacid homology of the in vitro tested IVE-TB antigens with BCG and other pathogenic mycobacteria.*

**Rv number**	**Amino acid homology (%)**			
	**BCG (taxid:33892)**	***M. leprae* (taxid:1769)**	***M. ulcerans* (taxid:1809)**	***M. kansasii* (taxid:1768)**
**Rv3875**	26%	64%	96%	99%
**Rv3874**	27%	16%	98%	97%
**Rv0287**	100%	84%	97%	96%
**Rv0288**	100%	85%	94%	95%
**Rv0383**	100%	87%	89%	91%
**Rv0423**	100%	94%	95%	97%
**Rv0440**	100%	96%	96%	99%
**Rv0467**	100%	32%	96%	96%
**Rv0468**	100%	94%	95%	95%
**Rv0470**	100%	91%	93%	93%
**Rv0501**	100%	82%	91%	90%
**Rv0640**	100%	94%	99%	96%
**Rv0642**	100%	94%	94%	95%
**Rv0645**	100%	93%	84%	94%
**Rv0824**	100%	90%	97%	93%
**Rv0826**	100%	11%	91%	91%
**Rv0867**	100%	29%	38%	38%
**Rv0991**	100%	55%	54%	53%
**Rv1009**	99%	86%	91%	91%
**Rv1038**	100%	76%	97%	98%
**Rv1131**	100%	48%	82%	80%
**Rv1221**	100%	84%	85%	87%
**Rv1284**	100%	7%	93%	94%
**Rv1390**	100%	95%	95%	91%
**Rv1479**	100%	94%	93%	93%
**Rv1733**	100%	7%	18%	49%
**Rv1737**	100%	5%	29%	86%
**Rv1738**	100%	15%	69%	64%
**Rv1791**	100%	51%	76%	85%
**Rv1792**	77%	Nssf	75%	75%
**Rv1846**	99%	86%	91%	91%
**Rv1872**	100%	91%	92%	93%
**Rv1980**	100%	5%	82%	83%
**Rv2007**	100%	62%	67%	80%
**Rv2029**	100%	10%	5%	78%
**Rv2031**	100%	28%	85%	89%
**Rv2032**	100%	10%	68%	71%
**Rv2034**	100%	13%	52%	86%
**Rv2215**	98%	84%	85%	86%
**Rv2245**	43%	41%	97%	97%
**Rv2346**	98%	76%	98%	97%
**Rv2347**	100%	74%	97%	98%
**Rv2382**	55%	54%	52%	79%
**Rv2389**	100%	51%	49%	62%
**Rv2431**	100%	46%	53%	49%
**Rv2450**	100%	35%	74%	83%
**Rv2461**	100%	97%	98%	97%
**Rv2626**	100%	16%	40%	92%
**Rv2710**	100%	96%	96%	97%
**Rv2873**	100%	18%	18%	84%
**Rv2941**	56%	55%	56%	89%
**Rv3048**	100%	97%	98%	98%
**Rv3052**	100%	80%	91%	92%
**Rv3353**	100%	13%	78%	80%
**Rv3407**	100%	5%	18%	91%
**Rv3462**	100%	100%	100%	100%
**Rv3583**	99%	99%	100%	100%
**Rv3614**	100%	82%	48%	71%
**Rv3615**	100%	68%	46%	75%
**Rv3616**	99%	77%	44%	73%
**Rv3846**	100%	88%	96%	99%
**Rv3865**	100%	41%	54%	89%

Nssf: no significant similarity found

Table S4. *IVE-TB proteins selected combining data from in vivo and in silico analyses.*

Subgroup	Criteria	List	Rv number	Expression rank (median RGCN)	Function	SNP non syn/protein length	n of alleles covered			DQ DP
							HLAIa	DR	>20 (sum)*	
I	Top 15% upregulated genes in last 6 wks *Mtb* infection	1	Rv2031	1 (157842,80)	HspX	99	9	16	**25**	12
		2	Rv3462	2 (102738,05)	IF-1 infA	100	8	16	**24**	9
		3	Rv1738	4 (77767,70)	Conserved hypothetical protein	99	8	17	**25**	8
		4	Rv3616	5 (75799,22)	EspA	98	12	17	**29**	12
		5	Rv3875	6 (69424,68)	ESAT6	99	6	13	19	6
		6	Rv3615	7 (68193,65)	EspC	98	10	16	**26**	9
		7	Rv0991	8 (63946,90)	Conserved serine rich protein	100	10	17	**27**	11
		8	Rv1221	9 (58858,66)	SigE	98	12	17	**29**	11
		9	Rv2007	10 (39200,24)	FdxA	99	7	17	**24**	12
		10	Rv2626	11 (38944,48)	Hrp1	99	12	17	**29**	11
		11	Rv2382	12 (37458,35)	Polyketide synthetase mbtC	100	12	17	**29**	11
		12	Rv1284	13 (36325,39)	Beta-carbonic anhydrase	99	11	17	**28**	10
		13	Rv2710	14 (34396,10)	SigB	100	12	17	**29**	12
		14	Rv3846	15 (32097,29)	SodA	98	12	17	**29**	12
		15	Rv0287	17 (26911,24)	EsxG	99	10	17	**27**	9
		16	Rv0288	18 (25482,65	EsxH	94	10	17	**27**	12
		17	Rv3052	19 (23525,29)	FadB4	100	12	17	**29**	9
		18	Rv1038c	22 (20241,80)	EsxJ	99	9	17	**26**	10
		19	Rv1792	26 (18245,72)	EsxM	100	9	17	**26**	10
		20	Rv2941	27 (16590,25)	28	99	12	17	**29**	12
		21	Rv3865	28 (16585,78)	EspF	97	12	17	**29**	9
		22	Rv0824c	29 (16455,57)	Acyl-desaturase DesA1	99	12	17	**29**	12

Subgroup	Criteria	List	Rv number	Expression rank (median RGCN)	Function	SNP non syn/protein length	n of alleles covered			DQ DP
							HLAIa	DR	>20 (sum)*	
II	Wide HLA coverage	23	Rv1131	39 (13339,33)	PrpC	98	12	17	**29**	12
		24	Rv0423c	51 (10506,68)	ThiC	99	12	17	**29**	12
		25	Rv0467	58 (9919,47)	icl1	100	12	17	**29**	12
		26	Rv0642c	59 (9805,62)	MmaA4	99	12	17	**29**	12
		27	Rv0826	137 (3223,96)	Conserved hypothetical protein	98	12	17	**29**	12
		28	Rv2245	56 (10230,41)	KasA	98	12	17	**29**	11
		29	Rv1980c	54 (10289,25)	Mpt64	99	12	17	**29**	10
		30	Rv0383c	38 (13406,05)	Possible conserved secreted protein	99	11	17	**28**	12
		31	Rv0440	45 (10783,72)	GroEL2	100	11	17	**28**	12
		32	Rv2873	53 (10342,89)	Mpt83	99	11	17	**28**	11
		33	Rv3583c	52 (10373,84)	Possible transcription factor	100	10	17	**27**	11
		34	Rv2347c	40 (12891,82)	EsxP	100	9	17	**26**	10
		35	Rv3407	44 (10870,06)	VapB47	99	9	16	**25**	9
		36	Rv1791	47 (10763,19)	PE19	100	9	15	**24**	10
		37	Rv2346c	48 (10718,72)	EsxO	99	7	16	**23**	10

Subgroup	Criteria	List	Rv number	NB/len Rank	Number of Binders (NB)	Length (len)	NB/len	Function
II	Top binders HLAIa	38	Rv3048c	12	105	324	0,3	R1F protein
		39	Rv2431c	14	31	99	0,3	PE25
		40	Rv2461	15	62	200	0,3	ClpP1
		23	Rv1131	18	121	393	0,3	PrpC
	Top binders HLAII	41	Rv0645	10	2479	286	8,7	MmaA1
		15	Rv0287	12	813	97	8,4	esxG
		42	Rv1479	14	3078	377	8,2	MoxR1
		40	Rv2461	16	1624	200	8,1	ClpP1

Subgroup	Criteria	List	Rv number	BCG (taxid:33892)	*M. leprae* (taxid:1769)	Function
III	Homology	43	Rv0468	100%	94%	FadB2
		44	Rv0470c	100%	91%	PcaA
		45	Rv0501	100%	82%	GalE2
		46	Rv0640	100%	94%	RplK
		47	Rv1390	100%	95%	RpoZ
		48	Rv1846	99%	86%	BlaI
		49	Rv1872	100%	91%	IldD2
		50	Rv2215	98%	84%	DlaT

In grey are the Rv number of proteins previously described in the literature as antigens; (sum)*: sum of predicted number of epitope binders for alleles HLA-Ia and DR.

Table S5. *E/C vs. IVE-TB antigens: comparison of the median cytokine fold-change of stimulated vs. unstimulated samples.*

	IFN-γ	IP-10	TNF-α	IL-17	IL-13	GM-CSF
Rv2382	□	□	□	□	□	■
Rv2431	□	□	□	□	□	■
Rv3407	□	□	□	□	□	■
Rv3846	□	□	□	□	□	■
Rv2245	□	□	□	□	□	■
Rv0066	□	□	□	□	□	□
Rv0383	□	□	□	□	■	■
Rv0824	□	□	□	□	□	■
Rv2710	□	□	□	□	□	■
Rv3583	□	□	□	□	□	□
Rv0501	□	□	□	□	□	■
Rv1479	□	□	□	□	□	■
Rv2031	□	□	□	□	■	■
Rv1038	□	□	□	□	■	■
Rv3462	□	□	□	□	□	■
E/C	▫	▫	▫	▫	▫	▫
Rv2007	□	□	□	▫	▫	■
Rv3052	□	□	□	■	□	■
Rv2941	□	□	□	■	■	■
Rv2215	□	□	□	■	□	■
Rv2873	□	□	□	■	□	■
Rv1738	□	□	□	■	■	□
Rv3048	□	□	□	■	□	■
Rv1792	□	□	□	■	□	■
Rv1390	□	□	□	■	□	■
Rv2346/47	□	□	□	■	■	■
Rv3614/15	□	□	□	■	□	■
Rv2626	□	□	□	■	■	■
Rv3615	□	■	□	■	■	□
Rv2657	□	■	□	■	■	■
Rv0640	□	■	□	□	■	■
Rv0287/88	■	□	□	■	■	■
Rv0423	□	■	▫	■	□	■
Rv1284	□	■	■	■	■	■
Rv0467	□	■	■	■	□	■
Rv0642	□	■	■	■	■	■
Rv1872	□	■	■	■	■	■
Rv0468	□	■	■	■	■	□
Rv0826	□	■	■	■	■	■
Rv0440	■	□	■	■	■	□
Rv1980	■	□	■	■	■	■
Rv0645	■	■	□	■	■	■
Rv3616	■	□	■	■	■	■
Rv1846	■	■	■	■	■	■
Rv1791	■	■	■	■	■	■
Rv0991	■	■	■	■	■	■
Rv2461	■	■	■	■	■	□
Rv0470	■	■	■	■	■	■
Rv1221	■	■	■	■	■	■
Rv1131	■	■	■	■	■	■
Rv3865	■	■	■	■	■	■
PPD	▒	▒	▒	▒	▒	□
PHA	▒	▒	▒	▒	▒	▒

Results based on multiplex assay performed with the supernatant of *Mtb*-exposed individuals (n = 12). Fold change similar to (▫), greater than (■), or less than (□) that induced by E/C; (▒): positive controls. E/C: ESAT6/CFP10.
